# Salivary MicroRNAs as Promising Biomarkers for Detection of Esophageal Cancer

**DOI:** 10.1371/journal.pone.0057502

**Published:** 2013-04-01

**Authors:** Zijun Xie, Gang Chen, Xuchao Zhang, Dongfeng Li, Jian Huang, Cuiqin Yang, Pingyong Zhang, Yuxuan Qin, Yifan Duan, Bo Gong, Zijun Li

**Affiliations:** 1 Department of Gastroenterology, Guangdong Academy of Medical Sciences, Guangdong General Hospital, Guangzhou City, Guangdong Province, China; 2 Graduate School, Southern Medical University, Guangzhou City, Guangdong Province, China; 3 Department of Thoracic Surgery, Guangzhou City, Guangdong Province, China; 4 Guangdong Lung Cancer Institute, Guangzhou City, Guangdong Province, China; 5 Research Center of Medical Sciences, Guangzhou City, Guangdong Province, China; 6 Physical Examination Center of East Ward, Guangzhou City, Guangdong Province, China; The Chinese University of Hong Kong, Hong Kong

## Abstract

**Background and Purpose:**

Tissue microRNAs (miRNAs) can detect cancers and predict prognosis. Several recent studies reported that tissue, plasma, and saliva miRNAs share similar expression profiles. In this study, we investigated the discriminatory power of salivary miRNAs (including whole saliva and saliva supernatant) for detection of esophageal cancer.

**Materials and Methods:**

By Agilent microarray, six deregulated miRNAs from whole saliva samples from seven patients with esophageal cancer and three healthy controls were selected. The six selected miRNAs were subjected to validation of their expression levels by RT-qPCR using both whole saliva and saliva supernatant samples from an independent set of 39 patients with esophageal cancer and 19 healthy controls.

**Results:**

Six miRNAs (miR-10b*, miR-144, miR-21, miR-451, miR-486-5p, and miR-634) were identified as targets by Agilent microarray. After validation by RT-qPCR, miR-10b*, miR-144, and miR-451 in whole saliva and miR-10b*, miR-144, miR-21, and miR-451 in saliva supernatant were significantly upregulated in patients, with sensitivities of 89.7, 92.3, 84.6, 79.5, 43.6, 89.7, and 51.3% and specificities of 57.9, 47.4, 57.9%, 57.9, 89.5, 47.4, and 84.2%, respectively.

**Conclusions:**

We found distinctive miRNAs for esophageal cancer in both whole saliva and saliva supernatant. These miRNAs possess discriminatory power for detection of esophageal cancer. Because saliva collection is noninvasive and convenient, salivary miRNAs show great promise as biomarkers for detection of esophageal cancer in areas at high risk.

## Introduction

Esophageal cancer (EC) is the eighth most common cancer and 6th leading cause of cancer mortality globally [1]. An estimated 482,300 new EC cases and 406,800 deaths occurred in 2008 worldwide. Incidence rates vary internationally by nearly 16-fold, with the highest rates in Southern and Eastern Africa and Eastern Asia and the lowest in Western and Middle Africa and Central America in both males and females. EC is 3 to 4 times more common among males than females [2]. Its incidence has increased rapidly in Western countries during the past half century [3]. The Chaoshan area of Guangdong Province in China has a high incidence of EC (>100/100,000) [4]. The death toll caused by EC in China accounts for more than 70% of all EC deaths worldwide [5].

The overall survival rate remains low; only 3–5% of diagnosed patients survive for 5 years [6]. In contrast, the survival rate increases to 90% in patients diagnosed with Stage I disease (T1N0M0) who undergo surgical resection [7]. Therefore, early diagnosis and treatment are vital. At present, the clinical diagnosis mainly depends on radiology and endoscopic biopsy. However, these tests are expensive, invasive, or cause discomfort to patients, and most patients are in a late stage of the disease when accurate diagnosis is attained. Therefore, it is necessary to identify a biomarker of early-stage EC.

Several studies have demonstrated that aberrant expression of miRNAs is closely related to the pathogenesis and development of cancer, and miRNAs possess discriminatory power as cancer biomarkers [8]. Several studies have reported that miRNAs are aberrantly expressed in cancer tissue and plasma in patients with EC [9], [10]. However, miRNA expression in the saliva of patients with EC has not yet been reported. Due to the extensive blood supply in salivary glands, saliva is considered to be a terminal product of blood circulation, and molecules that are present in plasma are also present in saliva. Hence, saliva is believed to mirror systemic health and reflect conditions such as cancers, infectious diseases, cardiovascular diseases, *etc*. [11]. Tissue, plasma, and saliva miRNAs share similar expression profiles [12]–[16]. This study comprised two phases: the discovery and validation phases. In the discovery phase, six miRNAs that were dysregulated most significantly in whole saliva of patients with EC by Agilent microarray analysis were selected as targets. In the validation phase, the expression levels of the six target miRNAs were validated by RT-qPCR using both whole saliva and saliva supernatant samples.

## Materials and Methods

### Sample size estimation

In the discovery phase, according to the Agilent microarray results, the sensitivity of miR-144 for EC was 85.7%. The formula for sample size estimation was as follows: n = *u_α_*_/2_P(1−P)/*δ^2^*.

In this formula, *n* is the number needed, *u_α_*_/2_ is the test level, *α is* the cutoff value of two-tailed normal distribution, *P* is the expected value of sensitivity, and *δ* is the permissible error.

According to the ability to attain the sample size for our study, we chose the values *α* = 0.05 (*u_α_*_/2_  = 1.96), *δ* = 0.11, and the values were substituted into the formula. The result was *n* = 1.96^2^ ×0.857× (1−0.857)/0.15^2^ ≈38.9 = 39. That is, in the validation phase of this study, the minimum number of cases needed for the EC group was 39, and 39 cases were chosen. According to the ability to attain the sample size for our study, we assumed the ratio of cases in the EC group to that in the healthy group to be 2∶1. 19 cases in healthy group were chosen.

### Subject selection

46 whole saliva and 46 saliva supernatant samples from 46 patients with EC and 22 whole saliva and 22 saliva supernatant samples from age-, gender-, and ethnically-matched healthy individuals were obtained from Guangdong General Hospital between July, 2011 and January, 2012. Patient histopathology results were confirmed by endoscopic biopsy, and the EC patients had no concomitant organic, systemic, or oral diseases, such as hepatitis, diabetes mellitus, *etc*, that could influence EC marker expression levels. Cancer staging was based on the UICC/TMN staging system [17]. Stages I, II, and III were based on the histopathology results after surgical resection. Stage IV was based on histopathology results of puncture biopsy of metastatic nodes or PET-CT results. “N/A (Not Available)” was assigned when patients refused further tests or treatments. Healthy subjects were collected as controls based on negative health examination results including blood tests, chest X-rays, oral examinations, abdominal ultrasound examinations, fecal occult-blood testing, and digital rectal examinations. None of these controls had been diagnosed with any type of malignancy either previous to the study or at the time of sample collection. This study was approved by the Institutional Review Board and Ethics Committee at Guangdong General Hospital. All participants were provided written consent for their information to be stored in the hospital database and used for research.

### Saliva collection

Subjects were asked to refrain from eating, drinking, smoking, and oral hygiene procedures for at least 2 hours before the collection. To stimulate glandular salivary flow, subjects received a 2% citric acid solution for application to the bilateral posterior lateral surfaces of the tongue with a cotton swab for 5 s every 30 s. The citric acid stimulation continued at 30-second intervals during the entire collection procedure. Up to 5 mL of saliva from each subject was collected in a 50-mL centrifuge tube. A total of 2 mL of saliva was removed from the tube as a whole saliva sample. The remaining 3 mL of saliva samples was centrifuged at 3,000× *g* for 15 min at 4°C to spin down exfoliated cells, and the supernatant was transferred into microcentrifuge tubes followed by a second centrifugation at 12,000× *g* for 10 min at 4°C to completely remove cellular components as saliva supernatant samples. Whole saliva is the saliva which has not been centrifuged. it may contain exfoliated esophageal cancer cells regurgitated from the esophagus due to the obstruction by esophageal tumor. Saliva supernatant is the saliva which has been centrifuged and it does not contain any exfoliated cells and other pellets. Hence saliva supernatant is considered to be the terminal product of blood circulation and it may reflect internal environment of our bodis. Samples were stored at −80°C until use. The procedure mentioned above must be finished within 2 h [18].

### Agilent microarray in discovery phase

**Figure 1:**
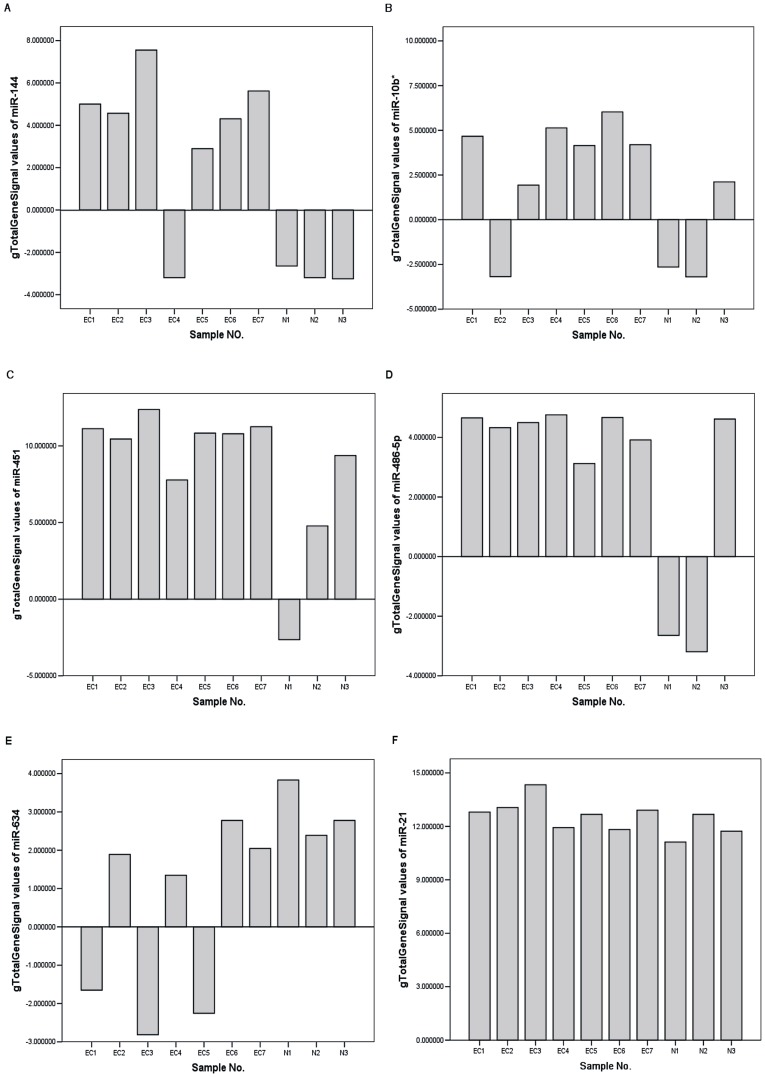
The bar charts of gTotalGeneSignal value of the six target miRNAs. A) With the exception of sample 4, the values of the six EC group samples were higher than those of the three healthy group samples. B) With the exception of sample 2, the values of the six EC group samples were higher than those of the three healthy group samples. C) With the exception of sample 10, the values of the seven EC group samples were higher than those of the two healthy group samples. D) With the exception of sample 10, the values of the seven EC group samples were higher than those of the two healthy group samples. E) All EC group values were lower than the three of the healthy group. F) Several studies have reported that miRNAs are aberrantly expressed in cancer tissue and plasma of EC patients; however, the gTotalGeneSignal value of miR-21 did not differ between the EC and healthy groups. However, it was also selected as a target miRNA.

Because this is the first research on salivary microRNAs for the detection of EC in the world, we just chose 10 cases to perform microarray. Seven whole saliva samples from the EC group and 3 from the healthy group were selected randomly. The pathology of all seven patients with EC was squamous cell carcinoma; one was stage I, one stage II, 3 stage III, and two stage IV. A total of 923 mature miRNA sequences were assembled and integrated into our miRNA microarray design. Raw data were normalized by Quantile algorithm, Gene Spring Software 11.0(Agilent technologies, Santa Clara, CA, US). 6 miRNAs were selected as targets, and their expression levels were validated by RT-qPCR in the validation phase. The selection method was as follows: the gTotalGeneSignal value in the Agilent microarray amounted to the expression level of each miRNA. Therefore, for identical miRNAs, bar charts were drawn according to the gTotalGeneSignal value of each miRNA. We selected five miRNAs: miR-144, miR-10b*, miR-451, miR-486-5p, and miR-634, the values of which tended to be much higher or lower in the EC group than in the healthy group and which also are closely associated with development of deseases. Meanwhile, several studies have reported that miR-21 is aberrantly expressed in cancer tissue and plasma from patients with EC [9], [10]; the gTotalGeneSignal value of miR-21 did not show this tendency, but was also selected as a target. The bar charts of the 6 target miRNAs were presented in Figure 1. (The seven bars in front represent the gTotalGeneSignal value from the EC group, and the 3 behind represent the gTotalGeneSignal value from the healthy group. Y-axis represents the value of gTotalGeneSignal).

### Validation phase

The expression levels of the 6 selected miRNAs were validated by RT-qPCR using both the 58 whole saliva samples and the 58 saliva supernatant samples from 39 patients with EC and 19 healthy controls. The mirVana PARIS Kit (Ambion, USA)was used to isolate total RNA from 1 mL of whole saliva or saliva supernatant, according to the manufacturer's protocol. Finally, RNA was eluted in 30 μL of preheated nuclease-free water (95°C) and stored at −80°C until use. The quality and concentration of isolated total RNA was determined using a NanodropND-1000 (Thermo Scientific, Worcester, MA), with the value by OD260/280 around 2.0 indicating high quality or purity. The reverse transcription reaction was first carried out with 11 μL of mixture containing 2 μL of RNA extract, 2 μL of RT primer (Ribo, China), and 7 μL of nuclease-free water. The 11-μL mixture was incubated at 70°C for 10 min and in ice for 2 min. Next, 5 μL of RT buffer, 2 μL of dNTP (2.5 mM), 0.5 μL of RNase inhibitor (40 U/μl), 0.5 μL of reverse transcriptase (200 U/μL), and 6 μL of nuclease-free water were added to the 11-μL mixture. The reverse transcription reaction continued at 42°C for 60 min, 70°C for 10 min, and 4°C for ∞. cDNA solution (3 mL) was amplified using 9 mL of SYBR Premix Ex Taq (TaKaRa, China), 2 μL of miRNA forward primer, 2 μL of miRNA backward primer, and 4 μL of nuclease-free water in a final volume of 20 mL. Quantitative PCR was run on a Biorad CFX96 2.1(Biorad Biosystems), and the reaction mixtures were incubated at 95°C for 2 min, followed by 50 cycles of 95°C for 5 s and 60°C for 10 s. At the end of the PCR cycles, melting curve analysis was performed to validate generation of the expected PCR product. The setting of melting curve was 65.0 to 95.0°C at increments of 0.5°C for 0.05 min + plate read. Each sample was analyzed in triplicate. All Ct values were <36. The expression levels of each target miRNA were normalized to that of miR-16. miR-16 is the most widely used internal control microRNA in body-fluid samples, including plasma [10], [19]. And most importantly, in the discovery phase and validation phase, our study demonstrated that miR-16 could stably express in saliva and work as an internal control for salivary miRNAs. The raw data in the discovery phase can be downloaded from the website of GEO: www.ncbi.nlm.nih.gov/geo/info/linking.html, and the accession number is GSE41268. The methodoloy, the raw data and pictures of the validation of miR-16 as an internal control in our research were presented in the Data S1. All expression levels were calculated utilizing the 2^−ΔΔCt^ method [20]. Briefly: sample^i^ΔCt_ target miR_  =  sample^i^Ct _target miR_-sample^i^Ct_miR-16_; sample^i^ΔΔCt_ target miR_  =  sample^i^ΔCt_ target miR_ – the mean value of ΔCt_ target miR_ of the healthy group.

### Statistical analysis

Expression levels of miRNAs and ages were compared using the Mann – Whitney U test or the Kruskall – Wallis H test. Genders were compared using the χ^2^ test. A multivariate logistic regression model was established for the 4 risk factors: smoking, alcohol intake, drinking or eating at hot temperatures, and Chaoshanese nationality. The odds ratios (ORs) of the 4 risk factors were calculated by forward LR. Each OR was compared using the χ^2^ test. Receiver-operating characteristics (ROC) curves were used to evaluate the discriminatory power of each miRNA for differentiation of patients and controls. The correlation of each miRNA expression level between whole saliva and saliva supernatant was analyzed by Spearman's correlation test. The differences in the discriminatory powers of target miRNAs were compared with the method of Delong using the MedCalc12.2.1 software. Other statistical analyses were performed with the SPSS software, version 13.0 (SPSS, Inc., Chicago, IL). A *p* value of <0.05 was considered to indicate statistical significance.

## Results

### Whole saliva profile

Ten whole saliva samples (7 from patients with EC and 3 from healthy controls) were evaluated by Agilent microarray. A total of 923 mature miRNA sequences were assembled and integrated into our miRNA microarray design. A total of 461 miRNAs were detected in whole saliva (the probe signal value of these miRNAs was present in at least one sample). Of these 461 miRNAs, 452 were detected in EC samples and 391 in healthy control samples. A total of 232 miRNAs were detected in all 10 samples. A total of 261 miRNAs were detected in all 7 EC samples, and 243 miRNAs in all 3 control samples. Twenty-five miRNAs showed significant differences in expression between the EC group and the healthy group. 6 miRNAs (miR-144, miR-10b*, miR-21, miR-451, miR-486-5p, and miR-634) were subjected to validation of their expression levels by RT-qPCR. The number of miRNAs detected in each sample was presented in Table 1.

**Table 1:** Number of miRNAs detected in each sample.

Sample No.	EC1	EC2	EC3	EC4	EC5	EC6	EC7	NC1	NC2	NC3	Average
Number of miRNAs	365	334	388	336	377	330	329	270	336	351	342

Note: EC, esophageal cancer; NC, normal control.

### Characteristics of EC patients and healthy controls in the validation phase

In the validation phase, 39 patients with EC were selected, 32 (82.1%) of whom had squamous cell carcinoma, 4 (10.3%) had adenocarcinoma, and 3 (7.7%) had small cell carcinoma. Cancer staging of the patients with EC was as follows: 0 (0%) were in stage I, 12 (30.8%) in stage II, 11 (28.2%) in stage III, 10 (23.6%) in stage IV, and 6 were unavailable. Data regarding EC patients and healthy controls is presented in Table 2. All patients with EC were >40 years of age, mainly from Chaoshan, Guangdong Province, reported drinking or eating at high temperatures, and alcohol drinkers, and most were male smokers. According to the logistic regression model, the ORs of Chaoshanese individuals and drinking or eating at a high temperature were statistically significant (41.984 and 31.594, respectively).

**Table 2:** Characteristics of EC patients and healthy controls.

Charateristics	EC (n = 39)	Healthy controls (n = 19)	*p* value
**Gender**			0.249
Male	31 (79.5%)	15 (78.9%)	
Female	8 (20.5%)	4 (21.1%)	
**Age**			0.619
Mean ± SD	59.7±9.0	55.9±8.8	
Median (range)	58 (46–88)	57 (42–77)	
**Ethnicity**			
Han Chinese	39 (100%)	19 (100%)	
**Chaoshanese**		OR = 41.984	*p* = 0.000
Yes	25 (64.1%)	1 (5.3%)	
No	14 (35.9%)	18 (94.7%)	
**Drinking/eating at high temperature^1^**		OR = 31.594	*p* = 0.002
Yes	29 (74.4%)	2 (10.5%)	
No	9 (25.6%)	17 (89.5%)	
**Smoking**			
Yes	30 (76.9%)	6 (31.6%)	
No	9 (23.1%)	13 (68.4%)	
**Alcoholic^2^**			
Yes	28 (71.8%)	3 (15.8%)	
No	11 (28.2%)	16 (84.2%)	

Note: 1. High temperature: >60°C. 2. According to the standard of mainland China, the definition of an alcoholic is as follows: male, ≥40 g/d; female, ≥20 g/d; consumption of alcohol (g)  =  volume of alcohol (mL) × concentration of alcohol (%) ×0.8.

### Discriminatory power of whole saliva and saliva supernatant miRNAs for EC

The expression levels of the 6 selected target miRNAs (miR-10b*, miR-144, miR-21, miR-451, miR-486-5p, and miR-634) were validated by RT-qPCR using both whole saliva and saliva supernatant samples. 3 miRNAs (miR-10b*, miR-144, and miR-451) in whole saliva from the EC group were significantly upregulated (*p* = 0.001, 0.012, and 0.002, respectively; fold-changes, 58.7, 45.6, and 42.4, respectively). The other 3 miRNAs (miR-21, miR-486-5p, and miR-634) showed no significant differences between the EC group and healthy group (*p* = 0.110, 0.078, and 0.157, respectively). ROC curves were established to evaluate the discriminatory power of whole saliva miR-10b*, miR-144, and miR-451; the AUCs were 0.762, 0.706, and 0.756, respectively. An optimum cutoff value is needed for the ROC curve to define the discriminatory power. When the Younden index (Younden index  =  sensitivity + specificity -1) reaches the maximum value, the corresponding cutoff value will be the optimum cutoff value [21].

The cutoff values of the 3 whole saliva miRNAs (miR-10b*, miR-144, and miR-451) were determined to be 0.74530111, 0.78131771, and 0.79964271, respectively. Therefore, their sensitivities for EC detection were 89.7, 92.3, and 84.6% and specificities were 57.9, 47.4, and 57.9%, respectively.

4 miRNAs (miR-10b*, miR-144, miR-21, and miR-451) were significantly upregulated in EC group saliva supernatants (*p* = 0.013, 0.036, 0.015, and 0.006, respectively; fold-changes, 4.5, 6.7, 9.7, and 5.4, respectively; AUCs, 0.702, 0.671, 0.698, and 0.725, respectively). The other two miRNAs (miR-486-5p and miR-634) showed no significant differences between the EC group and healthy group (*p* = 0.211 and 0.524, respectively). By calculating the Younden index, the cutoff values of the 4 saliva supernatant miRNAs (miR-10b*, miR-144, miR-21, and miR-451) were determined to be 1.05078691, 8.08243248, 0.88851054, and 8.48817219, respectively. Therefore, their sensitivities for detection of EC were 79.5, 43.6, 89.7, and 51.3% and specificities were 57.9, 89.5, 47.4, and 84.2%, respectively.

**Figure 2:**
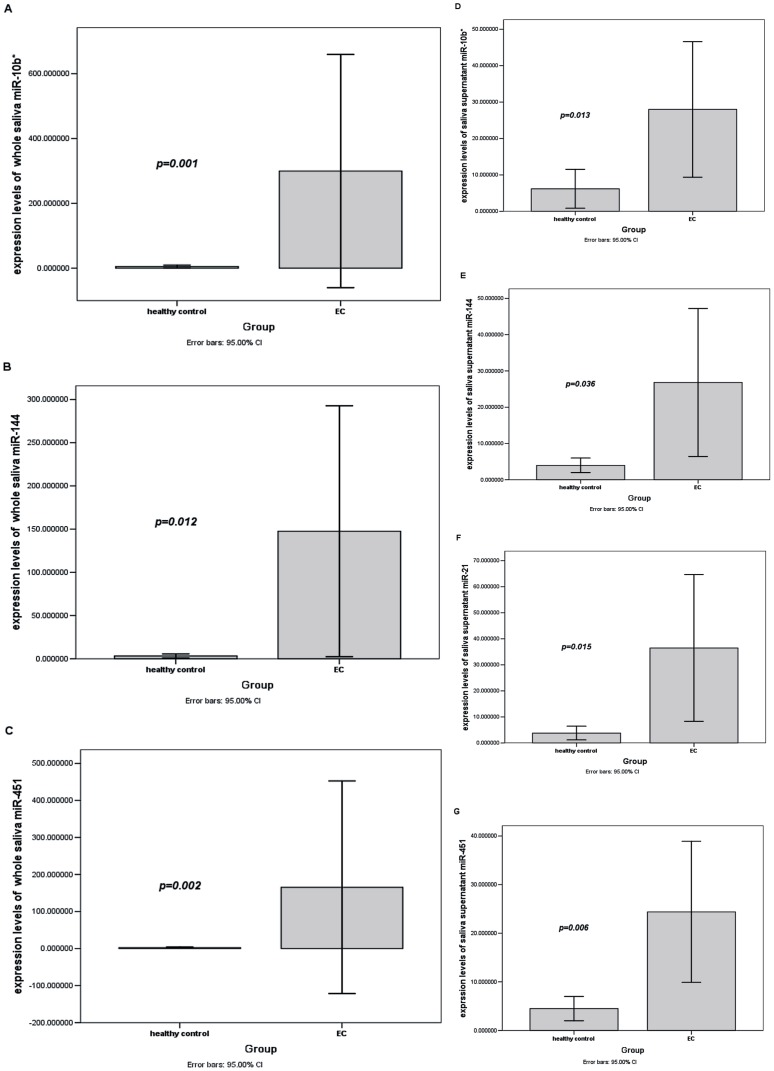
Bar charts of expression levels of aberrantly expressed miRNAs. **A**) whole saliva miR-10b*. **B**) whole saliva miR-144. **C**) whole saliva miR-451. **D**) saliva supernatant miR-10b*. **E**) saliva supernatant miR-144. **F**) saliva supernatant miR-21. **G**) saliva supernatant miR-451. miRNA expression levels were calculated by the 2^−ΔΔCt^ method. These miRNAs were significantly upregulated in the EC (n = 39) compared with the healthy (n = 19) group.

**Figure 3:**
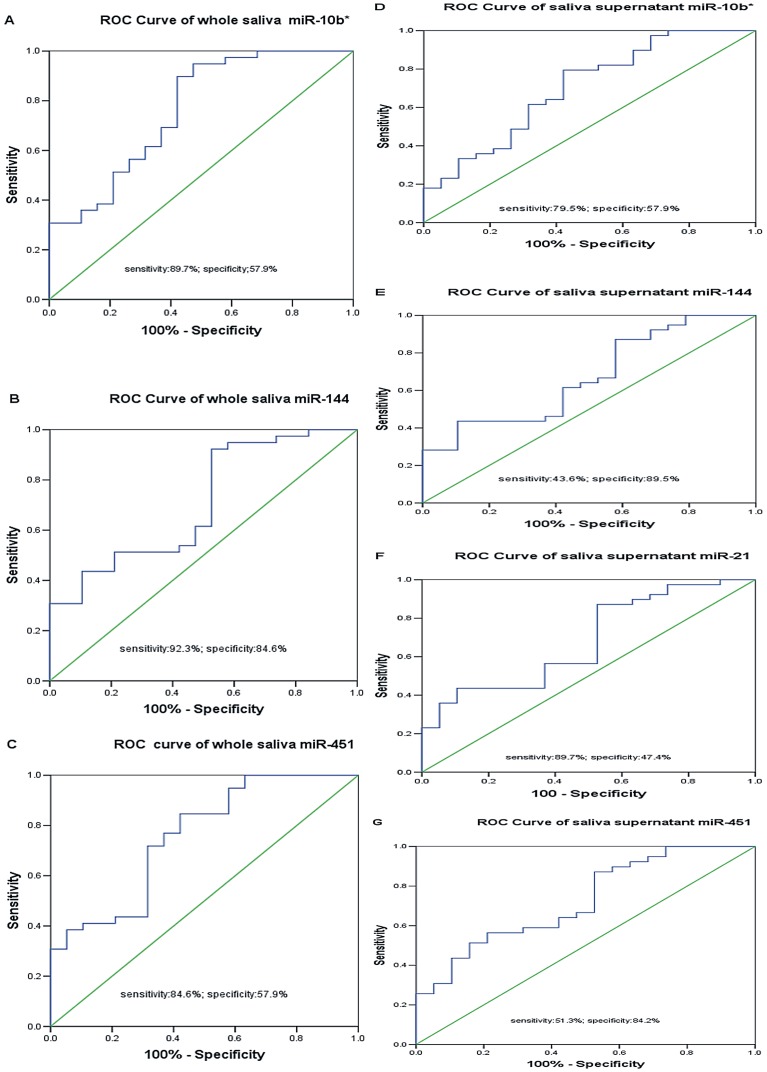
Receiver operating characteristic curve analysis for esophageal cancer diagnosis. A) Whole saliva miR-10b*. B)whole saliva miR-144, and C) whole saliva miR-451. D)saliva supernatant miR-10b*. E)saliva supernatant miR-144. F)saliva supernatant miR-21. G) saliva supernatant miR-451.

The raw data of the expression levels of each target miRNAs and the information of the research subjects in our study were presented in Data S2. The bar charts of expression levels of aberrantly expressed salivary miRNAs mentioned above were presented in Figure 2. The ROC curves of the aberrantly expressed miRNAs were presented in Figure 3.

### Comparison of AUCs

The larger the AUC, the seemingly more significant the discriminatory power. However, to diagnose a disease using the same collection of samples, it is not adequate to evaluate the discriminatory power simply by comparing AUC values [22], [23]. To accurately compare different AUCs, we used the method of Delong with the MedCalc software, version 12.2.1. 3 whole saliva and 4 saliva supernatant miRNAs were able to detect EC. miR-10b*, miR-144, and miR-451 could detect EC using both whole saliva and saliva supernatant. The AUCs indicated no significant differences among whole saliva miR-10b* and saliva supernatant miR-10b*, whole saliva miR-144 and saliva supernatant miR-144, and whole saliva miR-451 and saliva supernatant miR-451 (*p* = 0.3413, 0.7050, and 0.6867, respectively). The discriminatory powers of the same miRNAs in whole saliva and saliva supernatant were not significantly different. Furthermore, the AUCs of the 3 whole saliva miRNAs (miR-10b*, miR-144, and miR-451) that could detect EC were compared between two miRNAs. The results also showed no significant differences [*p* = 0.2356 (miR-10b* *vs*. miR-144), 0.8959 (miR-10b* *vs*. miR-451), and 0.3248 (miR-144 *vs*. miR-451)]. Finally, the AUCs of the 4 saliva supernatant miRNAs (miR-10b*, miR-144, miR-21, and miR-451) that could detect EC were compared between two miRNAs. The results also showed no significant differences [*p* = 0.8251 (miR-10b* *vs*. miR-21), 0.7699 (miR-10b* *vs*. miR-451), 0.7102 (miR-10b* *vs*. miR-144), 0.6079 (miR-21 *vs*. miR-451), 0.8729 (miR-21 *vs*. miR-144), and 0.3529 (miR-144 *vs*. miR-451)]. In conclusion, the discriminatory powers of all miRNAs in both whole saliva and saliva supernatant were similar.

### Correlation between whole saliva and saliva supernatant expression levels

Whole saliva and saliva supernatant samples were collected from each subject. According to the above results, miR-10b*, miR-144, and miR-451 were significantly upregulated in both whole saliva and saliva supernatant in the EC group. Spearman's correlation test was used to evaluate correlations of the expression levels of these 3 miRNAs between whole saliva and saliva supernatant. The expression levels of these 3 miRNAs in whole saliva and saliva supernatant showed significant correlations [*p* = 0.000 (miR-10b*), 0.035 (miR-144), and 0.003 (miR-451)].

### Relationships between miRNA expression levels and the clinical characteristics of EC patients

The expression levels of whole saliva miR-10b*, miR-144, and miR-451 and saliva supernatant miR-21, miR-10b*, miR-144, and miR-451 were not influenced by age, gender, residency, eating habits, smoking status, alcohol consumption, pathology type, cancer staging, cancer differentiation, or nodal metastasis of patients with EC (Table 3).

**Table 3:** Relationships between miRNA expression levels (means ± SD) and clinical characteristics of EC patients.

Feature	n	W^1^_miR-10b*	W_miR-144	W_miR-451	S_miR-10b*	S_miR-144	S_miR-21	S_miR-451
Gender		*p* = 0.945	*p* = 0.676	*p* = 0.889	*p* = 0.781	*p* = 0.126	*p* = 0.808	*p* = 0.465
Male	31	358±1238	184±498	206±993	26±52	24±67	21±40	30±68
Female	8	73±183	8±15	7±7	37±78	36±46	37±62	63±142
**Age (years)**		*p* = 0.593	*p* = 0.500	*p* = 694	*p* = 0.800	*p* = 0.448	*p* = 0.844	*p* = 0.715
≤61	20	261±992	116±382	37±130	34±75	44±84	32±59	61±117
>61	19	341±1247	181±517C	300±1266	21±31	8±12	16±21	10±12
**Chaoshanese**		*p* = 0.492	*p* = 0.708	*p* = 0.382	*p* = 0.553	*p* = 0.212	*p* = 0.190	*p* = 0.779
Yes	28	406±1300	178±519	203±1043	21±47	34±73	23±42	33±92
No	11	28±39	71±162	69±172	46±78	9±15	27±54	45±74
**Drinking or eating at high temperature**		*p* = 0.499	*p* = 0.898	*p* = 0.772	*p* = 0.797	*p* = 0.563	*p* = 0.274	*p* = 0.874
Yes	29	395±1278	71±170	198±1025	22±46	11±16	23±41	32±91
No	10	22±26	174±510	181±72	45±83	32±72	28±57	48±77
**Smoking**		*p* = 0.947	*p* = 0.351	*p* = 0.894	*p* = 0.424	*p* = 0.152	*p* = 0.527	*p* = 0.217
Yes	30	370±1258	90±505	213±1009	24±52	25±68	42±60	30±69
No	9	65±173	7±14	6±7	41±74	33±44	19±39	59±134
**Alcoholic**		*p* = 0.682	*p* = 0.482	*p* = 0.770	*p* = 0.501	*p* = 0.977	*p* = 0.639	*p* = 0.539
Yes	25	53±139	50±145	49±155	33±60	22±38	29±50	54±116
No	14	438±1372	202±545	231±1103	25±57	30±74	22±42	27±66
**Cancer staging**		*p* = 0.077	*p* = 0.149	*p* = 0.324	*p* = 0.107	*p* = 0.863	*p* = 0.324	*p* = 0.344
II	12	50±150	55±156	57±166	35±66	52±104	25±40	35±83
III	11	505±1643	194±632	505±1665	6±9	17±35	9±6	22±60
IV	10	100±181	120±311	15±18	41±81	15±27	45±73	67±133
N/A^2^	6							
**Nodal metastasis**		*p* = 0.131	*p* = 0.580	*p* = 0.658	*p* = 0.269	*p* = 0.632	*p* = 0.658	*p* = 0.357
Yes	12	50±150	55±156	57±166	35±66	52±104	25±40	35±83
No	21	411±1322	189±526	214±1062	25±54	16±29	24±47	37±90
N/A^2^	6							
**Pathology type**		*p* = 0.306	*p* = 0.789	*p* = 0.341	*p* = 0.107	*p* = 0.510	*p* = 0.798	*p* = 0.798
Squamous cell carcinoma	32	344±1219	129±462	181±975	31±69	19±42	21±39	36±92
Non-squamous cell carcinoma	7	95±222	230±395	93±218	9±11	68±96	39±67	37±67
**Differentiation**		*p* = 0.455	*p* = 0.969	*p* = 0.166	*p* = 0.653	*p* = 0.227	*p* = 0.399	*p* = 0.455
Moderate	14	11±16	46±145	48±155	57±82	32±65	16±30	40±89
Poor	7	87±195	15±36	11±15	12±19	71±110	53±65	77±149
Moderate-poor	5	1092±2440	422±937	1106±2471	24±46	7±11	12±23	9±16
N/A^2^	13							

Note:^1^W =  whole saliva, S =  saliva supernatant; ^2^N/A  =  not available due to patients' refusal of further tests or treatments; meanwhile, some patients in stage IV could not undergo operations and resected tumors were not available, so the differentiation of the tumors in these patients could not be determined.

## Discussion

miRNAs fall into two categories: cellular and extracellular. Extracellular miRNAs are present in plasma or other body fluids; they are also called secretory miRNAs. Several studies have found charateristic and stable miRNA profiles in bodily fluids. Extracellular miRNAs may be intercellular signaling molecules and transduct intercellular signals when flowing [24].

The mechanisms of how miRNA enters body fluids from cells are not clear. Kaosuka *et al*. [25] found that miRNA released from cells is regulated by neutral sphingomyelinase 2 (nSMase2). When ceramide increases in cells, nSMase2 promotes miRNA release. Inhibition of nSMase2 by the chemical inhibitor GW4869 impedes miRNA release from cells.

RNase is present in plasma, saliva, urine, and other body fluids. However, several studies have found that miRNAs in body fluids are stable and can resist degradation at high and low temperatures, in strong acids and bases, and by RNase. This is likely because free miRNAs in bodily fluids are wrapped up by proteins or stored within vesicles [26]–[28].

Saliva is a complex liquid that comprises secretions from the major and minor salivary glands. There are 450 to 750 minor accessory salivary glands situated on the tongue, buccal mucosa, and palate excluding the anterior part of the hard palate and gums [29]. There is also an extensive blood supply to these glands; therefore, molecules present in plasma are also present in saliva, such as proteins, DNA, RNA, *etc*. Several studies have reported that salivary molecules can detect oral and other organic and systemic diseases. Li *et al*. [30] reported that salivary mRNAs of DUSP1, H3F3A, OAZ1, S100P, SAT, IL-8, and IL-1 can detect oral cancer more accuately than can plasmatic mRNAs of these genes. Mbulaiteye *et al*. [31] found that salivary tests could replace blood tests to detect EB virus infection. Also, salivary C-erbB-2 and CA153 proteins could be new biomarkers of breast cancer [32]–[34].

Weber *et al*. [14] reported the miRNA profiles of 12 bodily fluids and found a maximum of 458 miRNAs in saliva. Patel *et al*. [35] found that the expression levels of salivary miRNAs were stable and the miRNA levels are reproducible within subjects. Park *et al*. [15] evaluated both whole saliva and saliva supernatant miRNAs in patients with oral cancer, and found similar miRNA profiles. They also reported that salivary miR-125a and miR-200a were significantly downregulated and that both might be new biomarkers of oral cancer.

By Agilent microarray, we detected the expression levels of 923 miRNAs in 10 whole saliva samples; 461 were detected. The expression levels of 25 miRNAs showed significant differences between the EC and healthy groups. Using the same technology, Weber *et al*.[14] detected 458 whole saliva miRNAs from 5 healthy subjects. After validation by RT-PCR, three whole saliva miRNAs (miR-10b*, miR-144, and miR-451) and four saliva supernatant miRNAs (miR-10b*, miR-144, miR-451, and miR-21) were significantly upregulated in patients with EC. Based on Spearman's correlation test, whole saliva and saliva supernatant miR-10b*, miR-144, and miR-451 showed significant correlations. The differences in AUCs were not statistically significant. This suggests that the discriminatory powers of whole saliva and saliva supernatant miRNAs were similar. However, the majority of patients with EC when admitted to the hospital showed symptoms of difficulty swallowing, regurgitation, or vomiting, and their saliva may have contained exfoliated EC cells regurgitated from the esophagus because of esophageal obstruction by the tumor. Therefore, if salivary miRNA testing is used to screen EC in asymptomatic patients, saliva supernatant is a better choice because the cellular components have been removed.

Biagioni F, *et al* [36] show that microRNA-10b* is a master regulator of breast cancer cell proliferation and is downregulated in tumoural samples versus matched peritumoural counterparts, they also suggests that restoration of microRNA-10b* expression might have therapeutic promise. But in our study, miR-10b* was upregulated in the saliva of EC patients. Because the origin of circulating miRNAs is not clear currently, their expression levels are not consistent with tissular or cellular miRNAs sometimes. For instance, Coulouarn *et al* [37] and Tsai *et al* [38] reported that miR-122 was downregulated in liver cancer tissue, but Zen and Zhang [39] reported that it was upregulated in the serum of patients with liver cancer. miR-10b* was upregulated in the saliva of EC patients, but downregulated in breast cancer tissue. The reason for it may be related to the different expression of miR-10b* in the two different kinds of cancer. In addition, it may be also associated with the different distribution between tissue and body fluids. Rossing *et al*. [40] found that miR-144 was downregulated in thyroid cancer tissue. Sureban *et al*. [41] reported that nanoparticle-based delivery of siDCAMKL-1 increased microRNA-144 and inhibited colorectal cancer tumor growth via a Notch-1-dependent mechanism. Konishi *et al*. [42] reported that the expression level of plasma miR-451 was increased in preoperative patients with gastric cancer and decreased postoperatively (AUC  = 0.96). Thus, their data suggest that plasma miR-451 possesses excellent discriminatory power for gastric cancer. Brenner *et al*. [43] found that upregulation of tissue miR-451 was predictive of a poor prognosis for gastric cancer patients. In contrast, Bandres *et al*. [44] reported that downregulation of miR-451 in gastric or colon cancer tissues predicted a poor prognosis. In addition, overexpression of miR-451 in gastric and colorectal cancer cells reduced cell proliferation and increased sensitivity to radiotherapy. Thus, their data suggest that miR-451 is an important tumor suppressor miRNA. Last but not least, Wang *et al* [45] reported that upregulated expression of miR-451 induced apoptosis and suppressed cell proliferation, invasion and metastasis in the esophageal carcinoma cell line EC9706. In addition, injection of miR-451 inhibited tumor growth in a xenograft model of esophageal cancer.

miR-144 and miR-451 are involved in the pathogenesis of myocardial ischemia and maintenance of erythroid homeostasis. Zhang *et al*. [46] reported that the synergistic effects of the GATA-4-mediated miR-144/451 cluster protected against simulated ischemia/reperfusion-induced cardiomyocyte death. Wang *et al*. [47] found that not only did the overexpression of miR-144/451 protect cardiomyocytes, but loss of the miR-144/451 cluster impairs ischemic preconditioning-mediated cardioprotection by targeting Rac-1. Rasmussen *et al*. [48], [49] reported that the miR-144/451eGFP allele could resolve the erythroid potential of hematopoietic precursors and that miR-451 had a greater impact on target gene expression than did miR-144. To-date, no study has found that miR-144 or miR-451 are aberrantly expressed in samples from EC patients. Our data suggest miR-144/451 involvement in the pathogenesis of EC.

miR-21 is closely related to various cancers, especially those of the gastrointestinal tract. Cancer tissue and plasma miR-21 were significantly upregulated in EC, gastric cancer, liver cancer, pancreatic cancer, bile duct cancer, colorectal cancer, *etc*. Furthermore, miR-21 may act as oncomir, and is closely associated with the pathogenesis, development, and metastasis of cancer. Recent studies have found that miR-21 is related to cancer target therapy, chemotherapy sensitivity, and drug resistance [50]. miR-21 was upregulated in both esophageal squamous cell carcinoma and adenocarcinoma tissue, which suggests that its expression level is not influenced by pathology type [51]. Komatsu *et al*. [10] reported that plasma miR-21 can detect esophageal squamous cell carcinoma and that patients with a high plasma miR-21 level tend to have greater vascular invasion and more frequent recurrence. Patients with Barrett's esophagus have a 30-fold higher risk of developing esophageal adenocarcinoma [52]. Smith *et al*. [53] reported that upregulation of miR-21 in the lesional epithelium of patients with Barrett's esophagus predicted development of esophageal adenocarcinoma. Therefore, miR-21 may be an appropriate marker in patients with Barrett's esophagus. In this study, whole saliva miR-21 was not upregulated in EC patients, but saliva supernatant miR-21 was upregulated significantly. This is possibly because the presence of normal exfoliated cells influences the levels of whole saliva miR-21. Meanwhile, it may be associated with the secretory mechanism of miR-21. In other words, miR-21 might not be upregulated in EC cancer tissues or cells [54], but due to the unknown mechanism, its expression level in circulation might be upregulated. Further studies are necessary in future to specify the secretory mechanism.

Therefore, if salivary miRNA testing is used to screen EC in asymptomatic patients, saliva supernatant is a better choice because the cellular components have been removed.

The Chaoshan area of Guangdong Province, China, has a high EC incidence (>100/100,000). The pathological types of EC are mainly squamous cell carcinoma and adenocarcinoma. More than 80% of EC cases in China are squamous cell carcinoma, but the majority in the West are adenocarcinoma [55]. The risk factors for EC in Chaoshan are smoking, alcohol consumption, and drinking or eating at high temperatures [56], [57]. The risk factors for EC in the West are gastroesophageal reflux disease and Barrett's esophagus [58]. In our study, a Chaoshanese ethnicity and drinking or eating at high temperatures were important risk factors (OR >30). The optimum temperature for beverages and foods is 10–40°C, and at temperatures >60°C, the esophageal epithelium is damaged and inflammation occurs. Chronic damage and inflammation may induce esophageal malignancy. Furthermore, high temperatures increase the assimilation of carcinogens such as nitrites within the body [4]. The majority of patients with EC in our study were from Chaoshan, who like to drink or eat at high temperatures. Also, Chaoshanese individuals possess several EC-risk genes, such as the AG phenotype in the CYP1A1 gene [59], *etc*. Thus risk genes and drinking and eating habits are related to the high incidence of EC in the Chaoshan area.

Saliva collection is more convenient, noninvasive, and cheaper than blood collection, so shows great promise in disease screening. We analyzed the expression levels of salivary miRNAs and investigated their EC discriminatory power to our knowledge for the first time worldwide. Our data suggest salivary miRNAs to be a new biomarker of EC. Functional studies to show the connection between significant microRNAs and EC are needed in our future researches. In addition, the expression levels of salivary miRNAs were not influenced by cancer staging, pathology type, differentiation, nodal metastasis, *etc*. Feber *et al* [51] reported that esophageal saquamous cell carcinoma and esophageal adenocarcinoma shared similar microRNA profile in tissue. Thus aberrant expression of miRNAs in saliva may be an early molecular event in the pathogenesis of EC and do not show heterogeneity in EC patients. Because detection of the expression levels of salivary miRNAs is convenient and noninvasive, it may be an option for early screening of EC. In conclusion, the role of salivary miRNAs in EC is worthy of further study.

## Supporting Information

Data S1 The methodoloy, the raw data and pictures of the validation of miR-16 as an internal control in our research. The methodoloy is in the first paragraph of the Word file, followed by an amplification curve of miR-16, Ct value of miR-16, and the concentration of tatal RNA of each sample from each research subjects.
(DOC)

Data S2 The raw data of the expression levels of each target miRNAs and the information of the research subjects in our study. All expression levels were calculated utilizing the 2^−ΔΔCt^ method. The information includes age, sex, ethnicity, Drinking/eating at high temperature, smoking status, alcoholic intake, Chaoshanese nationality, Differentiation, pathology types, node metastasis, cancer stage.
(XLS)
