# Supplementary material for: Salivary MicroRNAs as Promising Biomarkers for Detection of Esophageal Cancer
Source: PLoS One. 2013 Apr 1;8(4):e57502. doi: 10.1371/journal.pone.0057502 (PMC3613402; doi:10.1371/journal.pone.0057502)
Supplement: Data S1 — The methodoloy, the raw data and pictures of the validation of miR-16 as an internal control in our research. The methodoloy is in the first paragraph of the Word file, followed by an amplification curve of miR-16, Ct value of miR-16, and the concentration of tatal RNA of each sample from each research subjects. (DOC) [file pone.0057502.s001.doc]

**The methodoloy of the validation of miR-16 as an internal control in our research:**

In the validation phase, after the extraction of total RNA from each saliva samples, we measured the concentration of isolated total RNA using a NanodropND-1000. We assumed the concentration of each sample was Sample^i^Con. Next, we detected the expression level of miR-16 from each sample by RT-PCR. We assumed the Ct value of each sample was sample^i^Ct_miR-16_. Because the Ct value is inversely proportional to the expression level of miR-16, which means the higher the Ct value is, the lower expression level of miR-16 is.Therefore, we assumed a constant, 50, and subtracted sample^i^Ct_miR-16_ from 50,and the result we had was proportional to the expression level of miR-16.That is, the higher the result of 50-sample^i^Ct_miR-16_ is, the higher expression level of miR-16 is.At last, we assumed a variable V^i^ , and V^i^= Sample^i^Con/50-sample^i^Ct_miR-16_.So if the V^i^ of each sample remained stable, it represented the expression level of miR-16 in each individual from the 2 groups remained stable ,and miR-16 could be an internal control.According to this methodology,we calculated the V^i^ values of the whole saliva samples from 39 EC patients and 19 healthy controls, and the result demonstrated the expression level of miR-16 in each sample remained stable(*p*=0.709,Mann Whitney test),which meant that miR-16 could be an internal control in our study. The raw data and pictures were presented as follows:

Note:ECWS reprents whole saliva from EC patient; NWS reprents whole saliva from normal control. Sample^i^Ct_miR-16_. reprents the Ct value of miR-16 of each sample. Sample^i^Con reprents the concentration of each sample.

Amplification curve of miR-16:


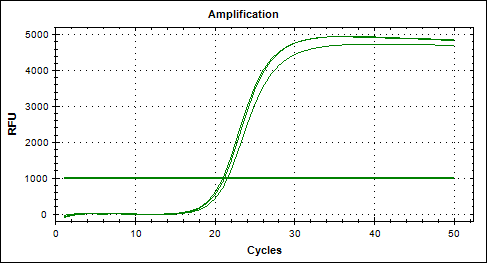


Ct value of miR-16:

| Well | Target | Sample | Cq | Cq Mean | Cq Std. Dev |
| --- | --- | --- | --- | --- | --- |
| A01 | mir16 | ECWS1 | 21.56 | 21.25 | 0.280 |
| A02 | mir16 | ECWS1 | 21.18 | 21.25 | 0.280 |
| A03 | mir16 | ECWS1 | 21.01 | 21.25 | 0.280 |

The concentration of Sample ECWS1:


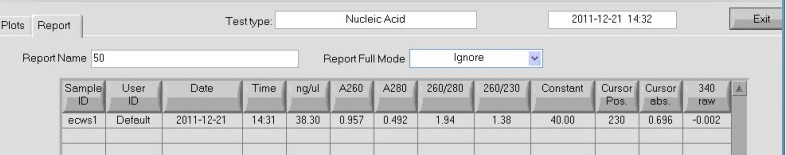


V ^ECWS1^= Sample ^ECWS1^Con /50-sample ^ECWS1^Ct_miR-16_

=38.3/50-21.25

=1.34386


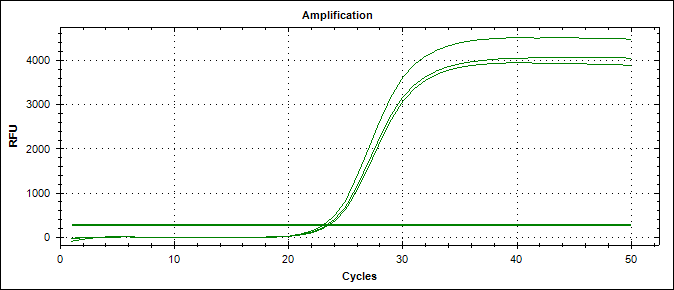


| Well | Target | Sample | Cq | Cq Mean | Cq Std. Dev |
| --- | --- | --- | --- | --- | --- |
| A01 | mir16 | ECWS2 | 23.47 | 23.26 | 0.235 |
| A02 | mir16 | ECWS2 | 23.29 | 23.26 | 0.235 |
| A03 | mir16 | ECWS2 | 23.00 | 23.26 | 0.235 |


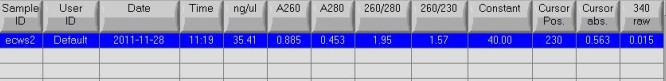


V ^ECWS2^= Sample ^ECWS2^Con /50-sample ^ECWS2^Ct_miR-16_

=35.41/26,74

=1.32423


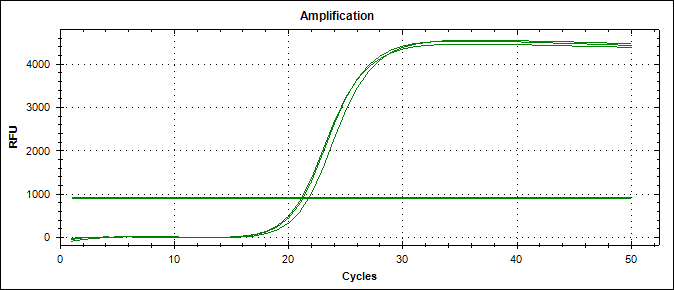


| Well | Target | Sample | Cq | Cq Mean | Cq Std. Dev |
| --- | --- | --- | --- | --- | --- |
| B01 | mir16 | ECWS3 | 21.74 | 21.37 | 0.332 |
| B02 | mir16 | ECWS3 | 21.24 | 21.37 | 0.332 |
| B03 | mir16 | ECWS3 | 21.12 | 21.37 | 0.332 |


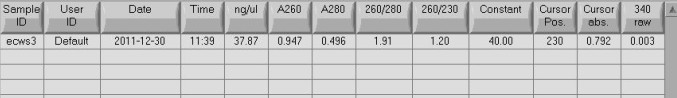


V ^ECWS3^= Sample ^ECWS3^Con /50-sample ^ECWS3^Ct_miR-16_

=37.87/28.63

=1.32274


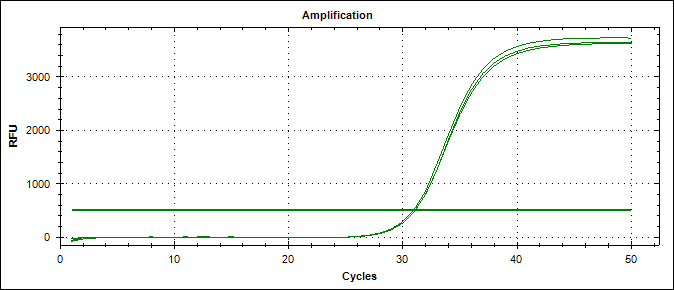


| Well | Target | Sample | Cq | Cq Mean | Cq Std. Dev |
| --- | --- | --- | --- | --- | --- |
| A01 | mir16 | ECWS4 | 31.53 | 31.49 | 0.086 |
| A02 | mir16 | ECWS4 | 31.54 | 31.49 | 0.086 |
| A03 | mir16 | ECWS4 | 31.39 | 31.49 | 0.086 |


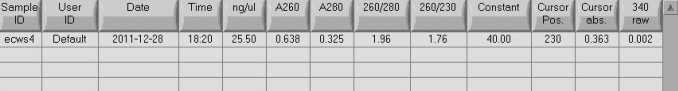


V ^ECWS4^= Sample ^ECWS4^Con /50-sample ^ECWS4^Ct_miR-16_

=25.5/18.51

=1.37763


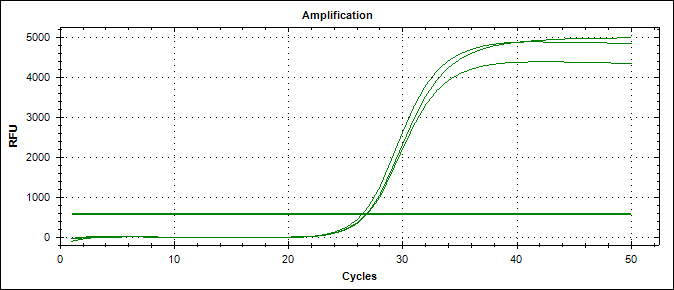


| Well | Target | Sample | Cq | Cq Mean | Cq Std. Dev |
| --- | --- | --- | --- | --- | --- |
| A01 | mir16 | ECWS5 | 26.91 | 26.73 | 0.243 |
| A02 | mir16 | ECWS5 | 26.82 | 26.73 | 0.243 |
| A03 | mir16 | ECWS5 | 26.45 | 26.73 | 0.243 |


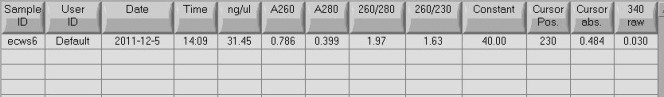


V ^ECWS5^= Sample ^ECWS5^Con /50-sample ^ECWS5^Ct_miR-16_

=31.45/23.27

=1.35153


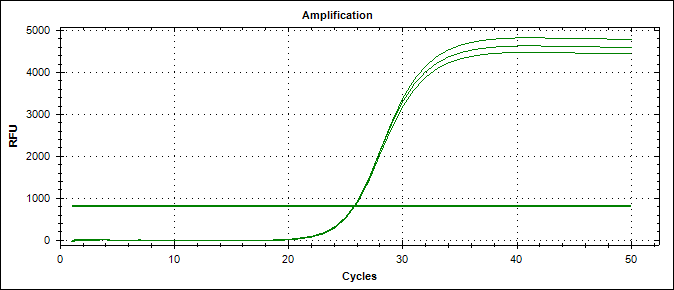


| Well | Target | Sample | Cq | Cq Mean | Cq Std. Dev |
| --- | --- | --- | --- | --- | --- |
| A04 | mir16 | ECWS6 | 25.81 | 25.79 | 0.054 |
| A05 | mir16 | ECWS6 | 25.83 | 25.79 | 0.054 |
| A06 | mir16 | ECWS6 | 25.73 | 25.79 | 0.054 |


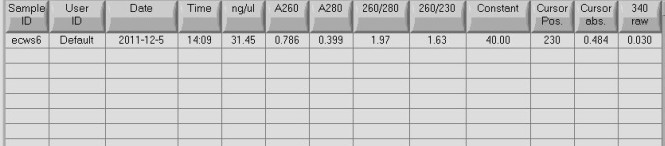


V ^ECWS6^= Sample ^ECWS6^Con /50-sample ^ECWS6^Ct_miR-16_

=31.45/24.21

=1.29905


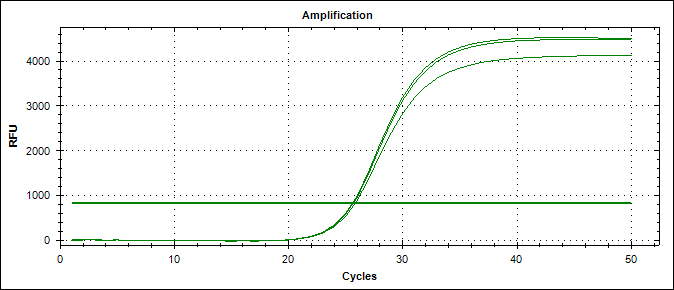


| Well | Target | Sample | Cq | Cq Mean | Cq Std. Dev |
| --- | --- | --- | --- | --- | --- |
| A04 | mir16 | ECWS7 | 25.62 | 25.72 | 0.126 |
| A05 | mir16 | ECWS7 | 25.69 | 25.72 | 0.126 |
| A06 | mir16 | ECWS7 | 25.86 | 25.72 | 0.126 |


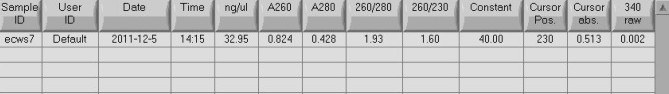


V ^ECWS7^= Sample ^ECWS7^Con /50-sample ^ECWS7^Ct_miR-16_

=32.95/24.28

=1.35708


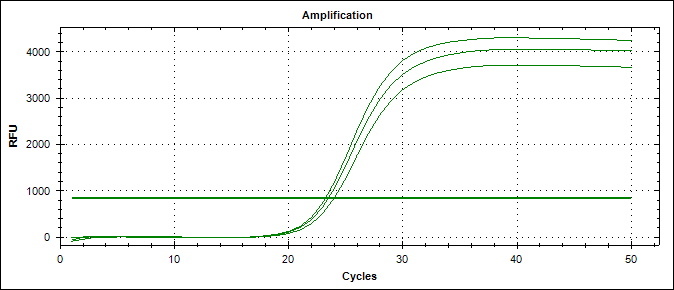


| Well | Target | Sample | Cq | Cq Mean | Cq Std. Dev |
| --- | --- | --- | --- | --- | --- |
| A01 | mir16 | ECWS8 | 23.99 | 23.57 | 0.387 |
| A02 | mir16 | ECWS8 | 23.49 | 23.57 | 0.387 |
| A03 | mir16 | ECWS8 | 23.23 | 23.57 | 0.387 |


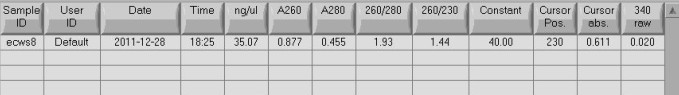


V ^ECWS8^= Sample ^ECWS8^Con /50-sample ^ECWS8^Ct_miR-16_

=35.07/26.43

=1.3269


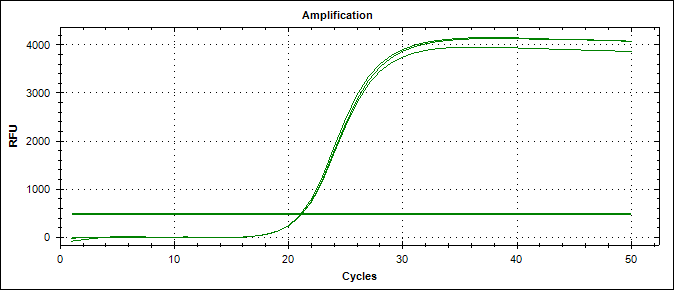


| Well | Target | Sample | Cq | Cq Mean | Cq Std. Dev |
| --- | --- | --- | --- | --- | --- |
| A01 | mir16 | ECWS9 | 21.18 | 21.14 | 0.050 |
| A02 | mir16 | ECWS9 | 21.15 | 21.14 | 0.050 |
| A03 | mir16 | ECWS9 | 21.08 | 21.14 | 0.050 |


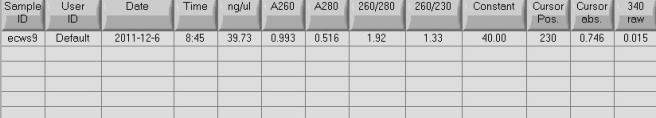


V ^ECWS9^= Sample ^ECWS9^Con /50-sample ^ECWS9^Ct_miR-16_

=39.73/28.86

=1.37665


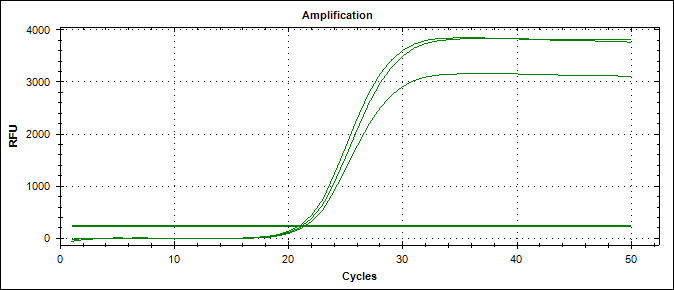


| Well | Target | Sample | Cq | Cq Mean | Cq Std. Dev |
| --- | --- | --- | --- | --- | --- |
| A01 | mir16 | ECWS10 | 23.37 | 23.06 | 0.309 |
| A02 | mir16 | ECWS10 | 23.06 | 23.06 | 0.309 |
| A03 | mir16 | ECWS10 | 22.75 | 23.06 | 0.309 |


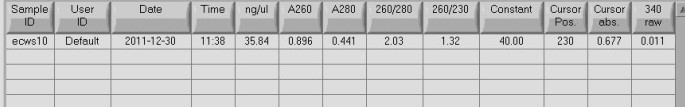


V ^ECWS10^= Sample ^ECWS10^Con /50-sample ^ECWS10^Ct_miR-16_

=35.87/26.94

=1.33148


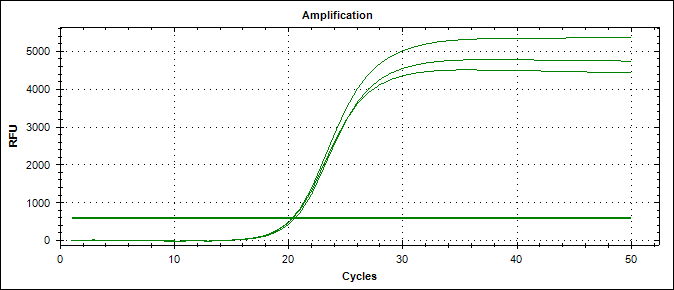


| Well | Target | Sample | Cq | Cq Mean | Cq Std. Dev |
| --- | --- | --- | --- | --- | --- |
| A04 | mir16 | ECWS11 | 20.51 | 20.38 | 0.120 |
| A05 | mir16 | ECWS11 | 20.28 | 20.38 | 0.120 |
| A06 | mir16 | ECWS11 | 20.34 | 20.38 | 0.120 |


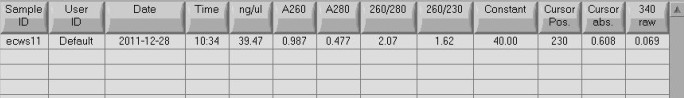


V ^ECWS11^= Sample ^ECWS11^Con /50-sample ^ECWS11^Ct_miR-16_

=39.47/29.62

=1.33255


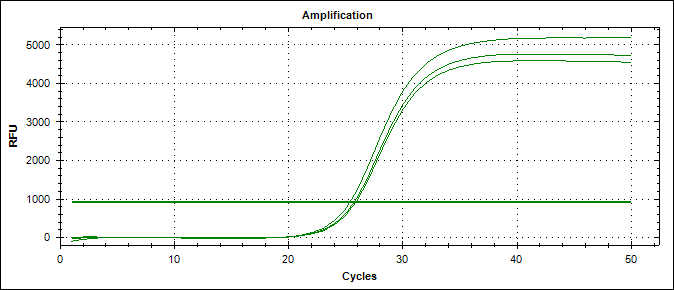


| Well | Target | Sample | Cq | Cq Mean | Cq Std. Dev |
| --- | --- | --- | --- | --- | --- |
| A01 | mir16 | ECWS12 | 25.90 | 25.66 | 0.275 |
| A02 | mir16 | ECWS12 | 25.73 | 25.66 | 0.275 |
| A03 | mir16 | ECWS12 | 25.36 | 25.66 | 0.275 |


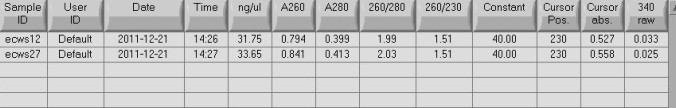


V ^ECWS12^= Sample ^ECWS12^Con /50-sample ^ECWS12^Ct_miR-16_

=31.75/24.34

=1.30444


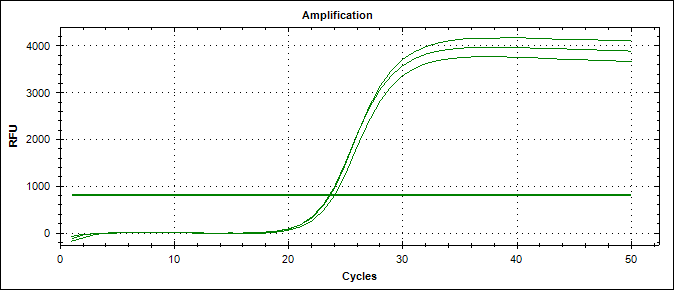


| Well | Target | Sample | Cq | Cq Mean | Cq Std. Dev |
| --- | --- | --- | --- | --- | --- |
| A01 | mir16 | ECWS13 | 24.06 | 23.77 | 0.253 |
| A02 | mir16 | ECWS13 | 23.67 | 23.77 | 0.253 |
| A03 | mir16 | ECWS13 | 23.59 | 23.77 | 0.253 |


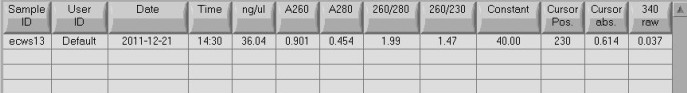


V ^ECWS13^= Sample ^ECWS13^Con /50-sample ^ECWS13^Ct_miR-16_

=36.04/26.23

=1.374


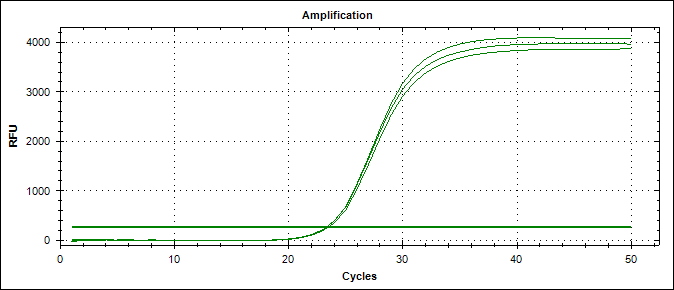


| Well | Target | Sample | Cq | Cq Mean | Cq Std. Dev |
| --- | --- | --- | --- | --- | --- |
| A04 | mir16 | ECWS14 | 23.51 | 23.39 | 0.107 |
| A05 | mir16 | ECWS14 | 23.33 | 23.39 | 0.107 |
| A06 | mir16 | ECWS14 | 23.32 | 23.39 | 0.107 |


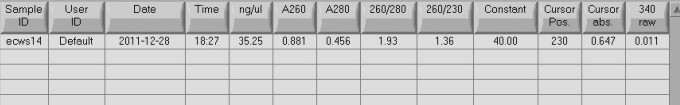


V ^ECWS14^= Sample ^ECWS14^Con /50-sample ^ECWS14^Ct_miR-16_

=35.25/26.61

=1.32469


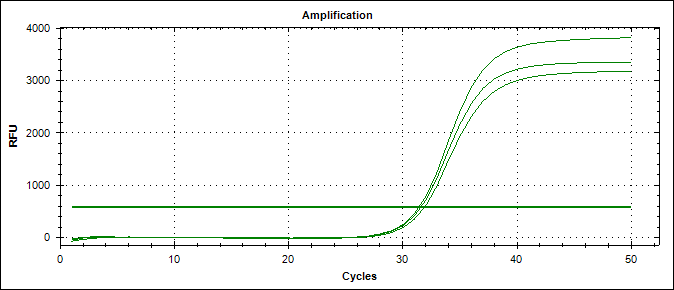


| Well | Target | Sample | Cq | Cq Mean | Cq Std. Dev |
| --- | --- | --- | --- | --- | --- |
| A01 | mir16 | ECWS15 | 31.91 | 31.63 | 0.259 |
| A02 | mir16 | ECWS15 | 31.58 | 31.63 | 0.259 |
| A03 | mir16 | ECWS15 | 31.40 | 31.63 | 0.259 |


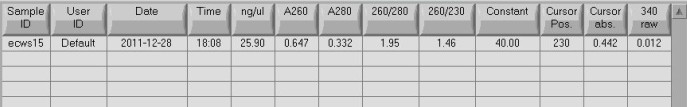


V ^ECWS15^= Sample ^ECWS15^Con /50-sample ^ECWS15^Ct_miR-16_

=25.9/18.37

=1.4099


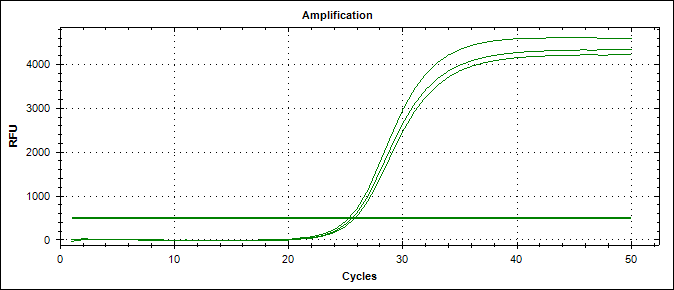


| Well | Target | Sample | Cq | Cq Mean | Cq Std. Dev |
| --- | --- | --- | --- | --- | --- |
| A04 | mir16 | ECWS16 | 26.08 | 26.06 | 0.261 |
| A05 | mir16 | ECWS16 | 26.31 | 26.06 | 0.261 |
| A06 | mir16 | ECWS16 | 25.79 | 26.06 | 0.261 |


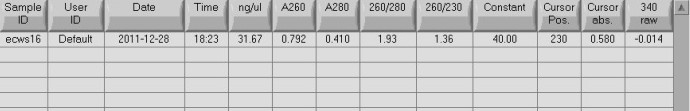


V ^ECWS16^= Sample ^ECWS16^Con /50-sample ^ECWS16^Ct_miR-16_

=31.67/23.94

=1.32289


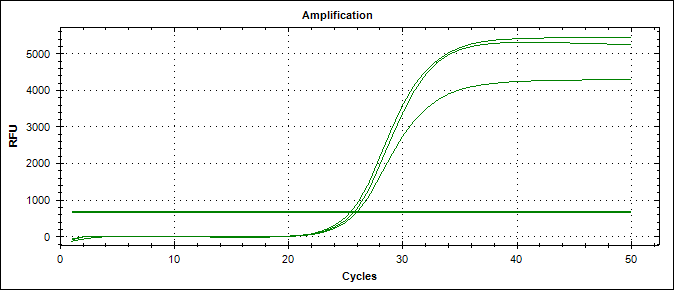


| Well | Target | Sample | Cq | Cq Mean | Cq Std. Dev |
| --- | --- | --- | --- | --- | --- |
| A01 | mir16 | ECWS17 | 25.67 | 25.68 | 0.314 |
| A02 | mir16 | ECWS17 | 26.00 | 25.68 | 0.314 |
| A03 | mir16 | ECWS17 | 25.37 | 25.68 | 0.314 |


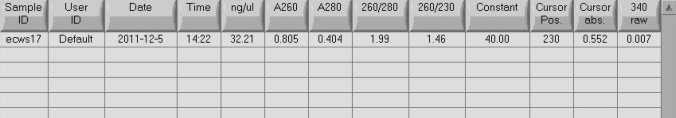


V ^ECWS17^= Sample ^ECWS17^Con /50-sample ^ECWS17^Ct_miR-16_

=32.21/24.32

=1.32442


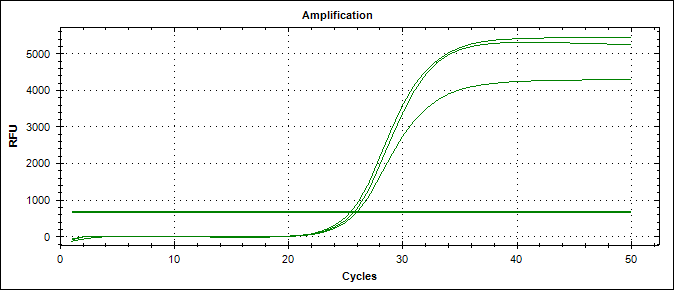


| Well | Target | Sample | Cq | Cq Mean | Cq Std. Dev |
| --- | --- | --- | --- | --- | --- |
| A04 | mir16 | ECWS18 | 25.25 | 25.35 | 0.301 |
| A05 | mir16 | ECWS18 | 25.69 | 25.35 | 0.301 |
| A06 | mir16 | ECWS18 | 25.12 | 25.35 | 0.301 |


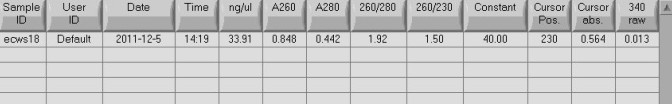


V ^ECWS18^= Sample ^ECWS18^Con /50-sample ^ECWS11^Ct_miR-16_

=33.91/24.65

=1.37566


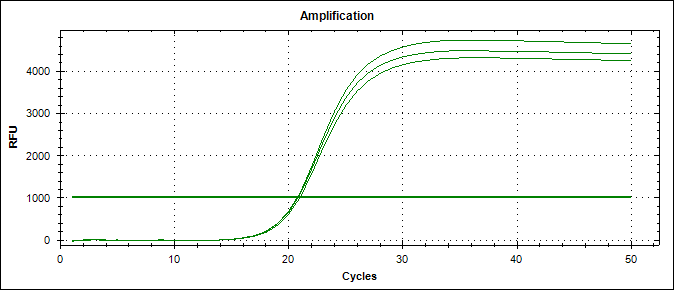


| Well | Target | Sample | Cq | Cq Mean | Cq Std. Dev |
| --- | --- | --- | --- | --- | --- |
| A04 | mir16 | ECWS19 | 20.75 | 20.86 | 0.132 |
| A05 | mir16 | ECWS19 | 20.82 | 20.86 | 0.132 |
| A06 | mir16 | ECWS19 | 21.00 | 20.86 | 0.132 |


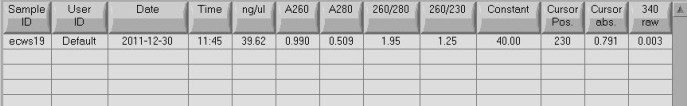


V ^ECWS19^= Sample ^ECWS19^Con /50-sample ^ECWS19^Ct_miR-16_

=39.62/29.14

=1.35964


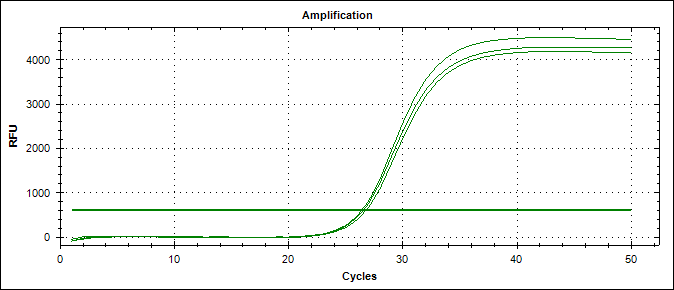


| Well | Target | Sample | Cq | Cq Mean | Cq Std. Dev |
| --- | --- | --- | --- | --- | --- |
| A01 | mir16 | ECWS20 | 26.77 | 26.56 | 0.196 |
| A02 | mir16 | ECWS20 | 26.51 | 26.56 | 0.196 |
| A03 | mir16 | ECWS20 | 26.39 | 26.56 | 0.196 |


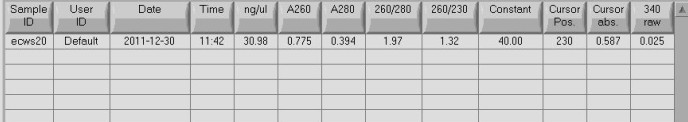


V ^ECWS20^= Sample ^ECWS20^Con /50-sample ^ECWS20^Ct_miR-16_

=30.98/23.44

=1.32167


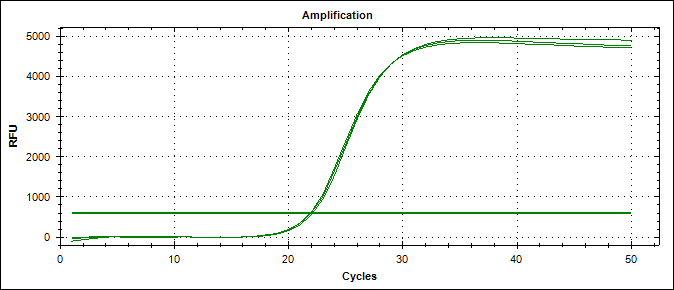


| Well | Target | Sample | Cq | Cq Mean | Cq Std. Dev |
| --- | --- | --- | --- | --- | --- |
| A01 | mir16 | ECWS21 | 22.12 | 22.00 | 0.118 |
| A02 | mir16 | ECWS21 | 21.97 | 22.00 | 0.118 |
| A03 | mir16 | ECWS21 | 21.89 | 22.00 | 0.118 |


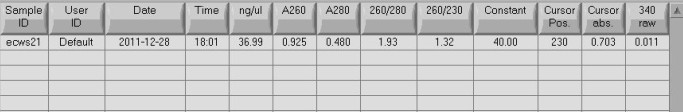


V ^ECWS21^= Sample ^ECWS21^Con /50-sample ^ECWS21^Ct_miR-16_

=36.99/28

=1.32107


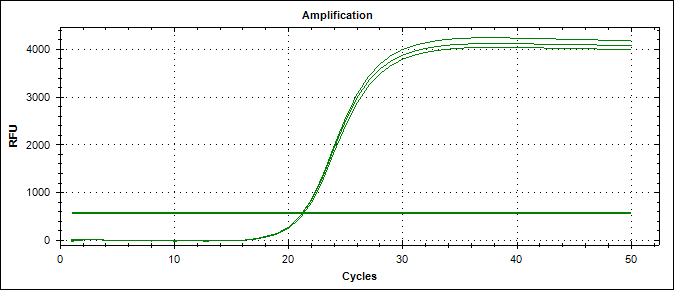


| Well | Target | Sample | Cq | Cq Mean | Cq Std. Dev |
| --- | --- | --- | --- | --- | --- |
| A04 | mir16 | ECWS22 | 21.36 | 21.27 | 0.079 |
| A05 | mir16 | ECWS22 | 21.22 | 21.27 | 0.079 |
| A06 | mir16 | ECWS22 | 21.24 | 21.27 | 0.079 |


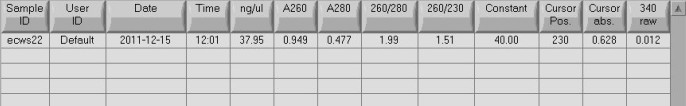


V ^ECWS22^= Sample ^ECWS22^Con /50-sample ^ECWS22^Ct_miR-16_

=37.95/28.73

=1.32092


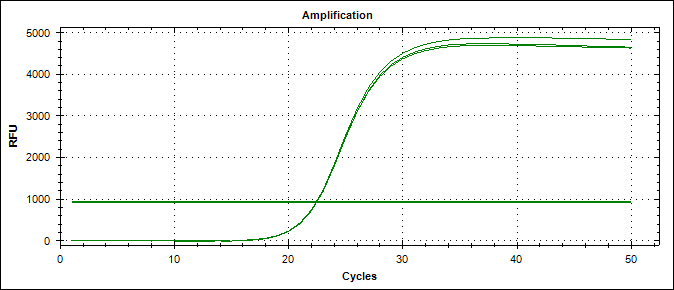


| Well | Target | Sample | Cq | Cq Mean | Cq Std. Dev |
| --- | --- | --- | --- | --- | --- |
| A04 | mir16 | ECWS23 | 22.45 | 22.41 | 0.037 |
| A05 | mir16 | ECWS23 | 22.38 | 22.41 | 0.037 |
| A06 | mir16 | ECWS23 | 22.39 | 22.41 | 0.037 |


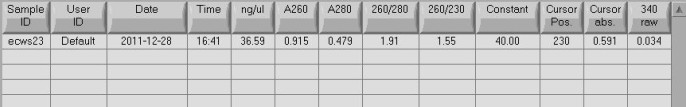


V ^ECWS23^= Sample ^ECWS23^Con /50-sample ^ECWS23^Ct_miR-16_

=36.59/27.59

=1.32621


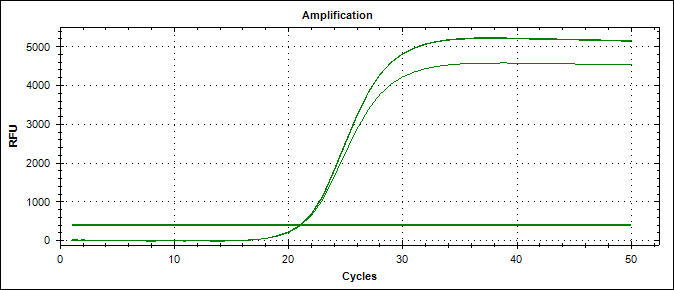


| Well | Target | Sample | Cq | Cq Mean | Cq Std. Dev |
| --- | --- | --- | --- | --- | --- |
| A04 | mir16 | ECWS24 | 21.01 | 21.04 | 0.043 |
| A05 | mir16 | ECWS24 | 21.02 | 21.04 | 0.043 |
| A06 | mir16 | ECWS24 | 21.09 | 21.04 | 0.043 |


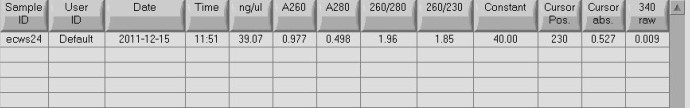


V ^ECWS24^= Sample ^ECWS24^Con /50-sample ^ECWS24^Ct_miR-16_

=39.07/28.86

=1.35378


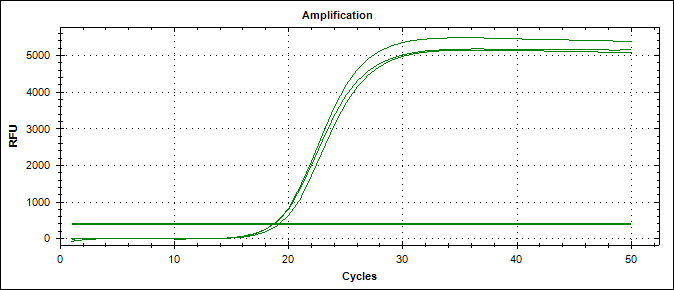


| Well | Target | Sample | Cq | Cq Mean | Cq Std. Dev |
| --- | --- | --- | --- | --- | --- |
| B01 | mir16 | ECWS25 | 19.17 | 18.85 | 0.279 |
| B02 | mir16 | ECWS25 | 18.67 | 18.85 | 0.279 |
| B03 | mir16 | ECWS25 | 18.71 | 18.85 | 0.279 |


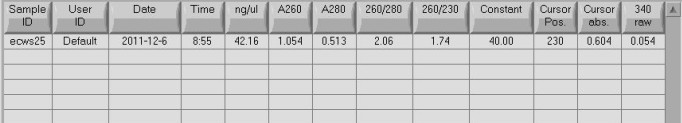


V ^ECWS25^= Sample ^ECWS25^Con /50-sample ^ECWS25^Ct_miR-16_

=42.16/31.15

=1.35345


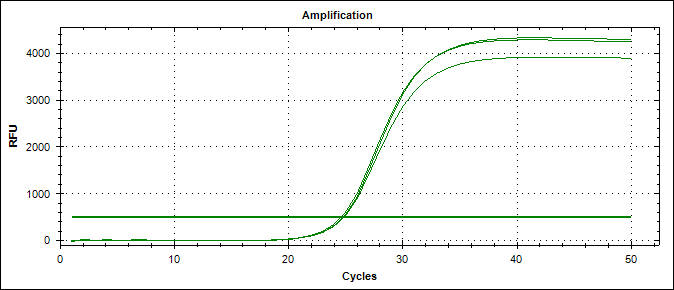


| Well | Target | Sample | Cq | Cq Mean | Cq Std. Dev |
| --- | --- | --- | --- | --- | --- |
| A04 | mir16 | ECWS26 | 24.71 | 24.69 | 0.132 |
| A05 | mir16 | ECWS26 | 24.55 | 24.69 | 0.132 |
| A06 | mir16 | ECWS26 | 24.81 | 24.69 | 0.132 |


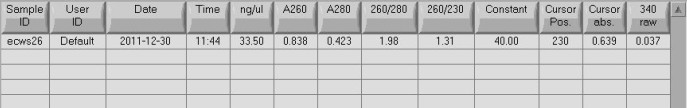


V ^ECWS26^= Sample ^ECWS26^Con /50-sample ^ECWS26^Ct_miR-16_

=33.5/25.31

=1.32359


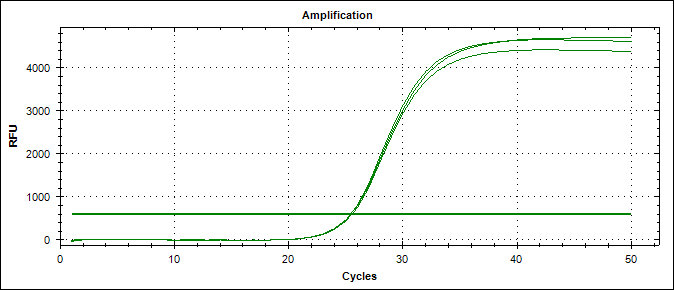


| Well | Target | Sample | Cq | Cq Mean | Cq Std. Dev |
| --- | --- | --- | --- | --- | --- |
| A04 | mir16 | ECWS27 | 25.43 | 25.47 | 0.071 |
| A05 | mir16 | ECWS27 | 25.55 | 25.47 | 0.071 |
| A06 | mir16 | ECWS27 | 25.44 | 25.47 | 0.071 |


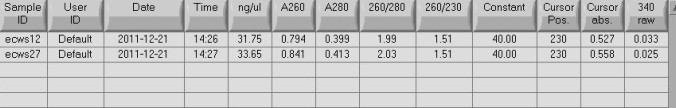


V ^ECWS27^= Sample ^ECWS27^Con /50-sample ^ECWS27^Ct_miR-16_

=33.65/24.53

=1.37179


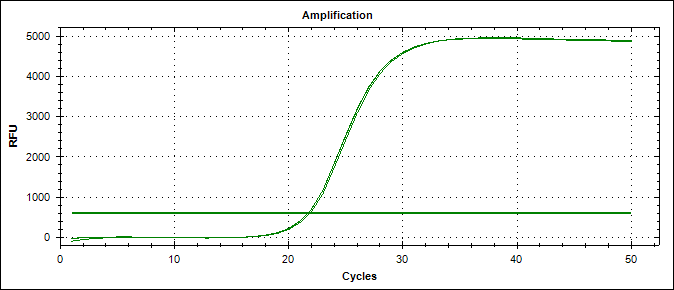


| Well | Target | Sample | Cq | Cq Mean | Cq Std. Dev |
| --- | --- | --- | --- | --- | --- |
| B01 | mir16 | ECWS28 | 21.85 | 21.71 | 0.117 |
| B02 | mir16 | ECWS28 | 21.64 | 21.71 | 0.117 |
| B03 | mir16 | ECWS28 | 21.65 | 21.71 | 0.117 |


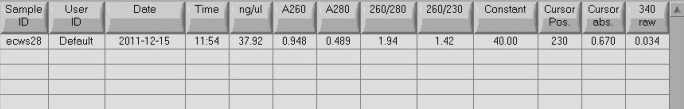


V ^ECWS28^= Sample ^ECWS28^Con /50-sample ^ECWS28^Ct_miR-16_

=37.92/28.29

=1.3404


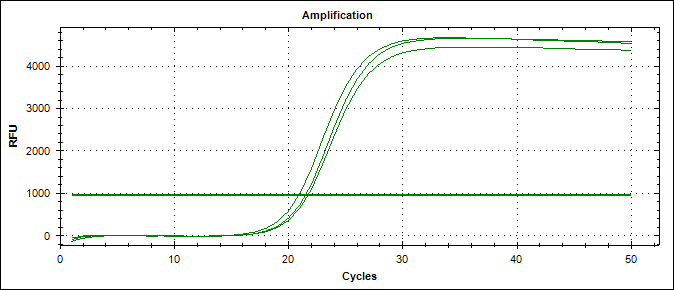


| Well | Target | Sample | Cq | Cq Mean | Cq Std. Dev |
| --- | --- | --- | --- | --- | --- |
| A01 | mir16 | ECWS29 | 21.68 | 21.34 | 0.411 |
| A02 | mir16 | ECWS29 | 21.46 | 21.34 | 0.411 |
| A03 | mir16 | ECWS29 | 20.88 | 21.34 | 0.411 |


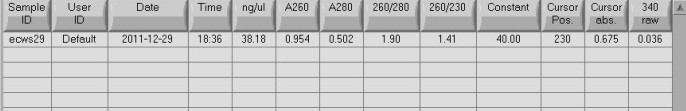


V ^ECWS29^= Sample ^ECWS29^Con /50-sample ^ECWS29^Ct_miR-16_

=38.18/28.66

=1.33217


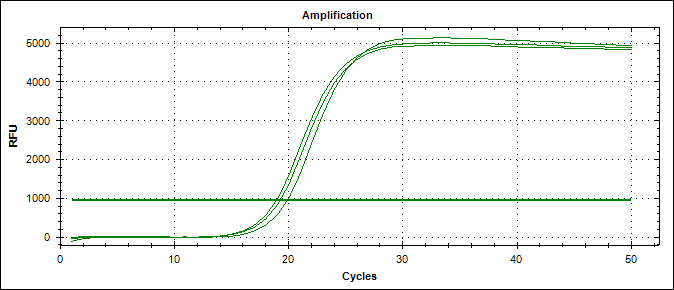


| Well | Target | Sample | Cq | Cq Mean | Cq Std. Dev |
| --- | --- | --- | --- | --- | --- |
| B01 | mir16 | ECWS30 | 19.91 | 19.37 | 0.487 |
| B02 | mir16 | ECWS30 | 19.26 | 19.37 | 0.487 |
| B03 | mir16 | ECWS30 | 18.96 | 19.37 | 0.487 |


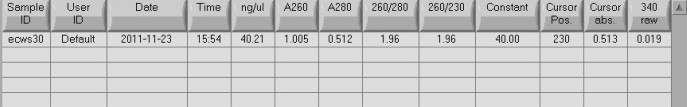


V ^ECWS30^= Sample ^ECWS30^Con /50-sample ^ECWS30^Ct_miR-16_

=40.21/30.63

=1.31277


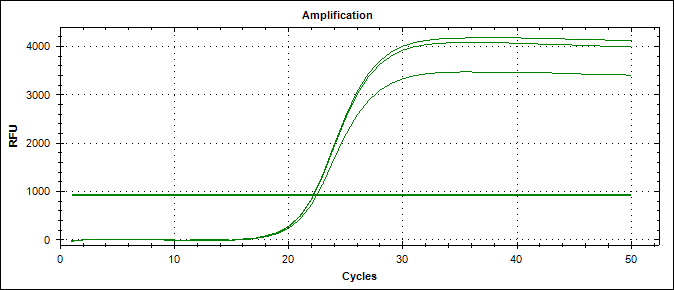


| Well | Target | Sample | Cq | Cq Mean | Cq Std. Dev |
| --- | --- | --- | --- | --- | --- |
| A04 | mir16 | ECWS31 | 22.18 | 22.26 | 0.164 |
| A05 | mir16 | ECWS31 | 22.15 | 22.26 | 0.164 |
| A06 | mir16 | ECWS31 | 22.45 | 22.26 | 0.164 |


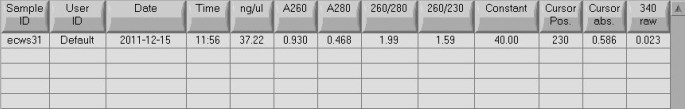


V ^ECWS31^= Sample ^ECWS31^Con /50-sample ^ECWS31^Ct_miR-16_

=37.22/27.74

=1.34174


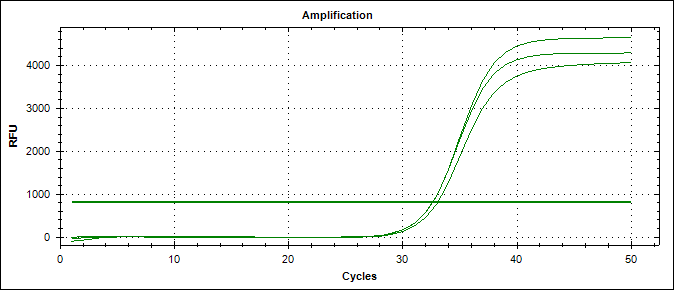


| Well | Target | Sample | Cq | Cq Mean | Cq Std. Dev |
| --- | --- | --- | --- | --- | --- |
| B01 | mir16 | ECWS32 | 32.61 | 32.77 | 0.267 |
| B02 | mir16 | ECWS32 | 32.62 | 32.77 | 0.267 |
| B03 | mir16 | ECWS32 | 33.08 | 32.77 | 0.267 |


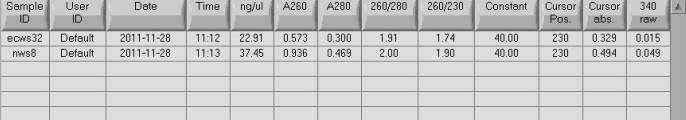


V ^ECWS32^= Sample ^ECWS32^Con /50-sample ^ECWS32^Ct_miR-16_

=22.91/17.23

=1.32966


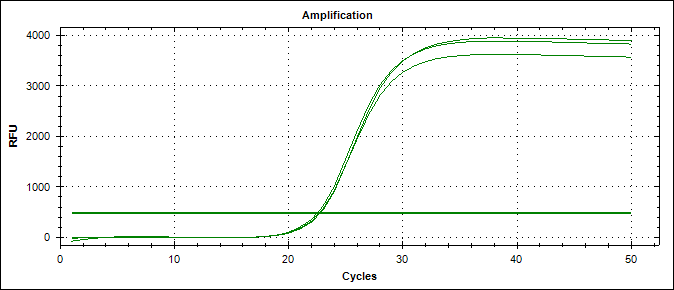


| Well | Target | Sample | Cq | Cq Mean | Cq Std. Dev |
| --- | --- | --- | --- | --- | --- |
| B01 | mir16 | ECWS33 | 22.81 | 22.68 | 0.144 |
| B02 | mir16 | ECWS33 | 22.71 | 22.68 | 0.144 |
| B03 | mir16 | ECWS33 | 22.52 | 22.68 | 0.144 |


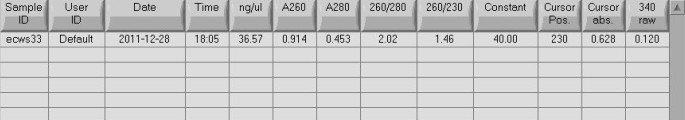


V ^ECWS33^= Sample ^ECWS33^Con /50-sample ^ECWS33^Ct_miR-16_

=36.57/27.32

=1.33858


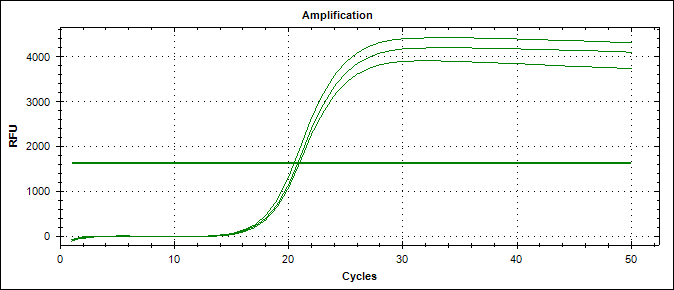


| Well | Target | Sample | Cq | Cq Mean | Cq Std. Dev |
| --- | --- | --- | --- | --- | --- |
| A01 | mir16 | ECWS34 | 20.95 | 20.74 | 0.244 |
| A02 | mir16 | ECWS34 | 20.79 | 20.74 | 0.244 |
| A03 | mir16 | ECWS34 | 20.47 | 20.74 | 0.244 |


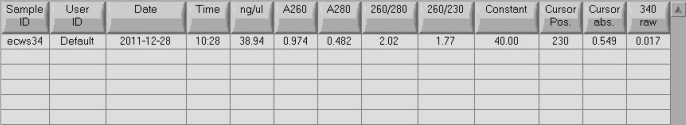


V ^ECWS34^= Sample ^ECWS34^Con /50-sample ^ECWS34^Ct_miR-16_

=38.94/28.26

=1.37792


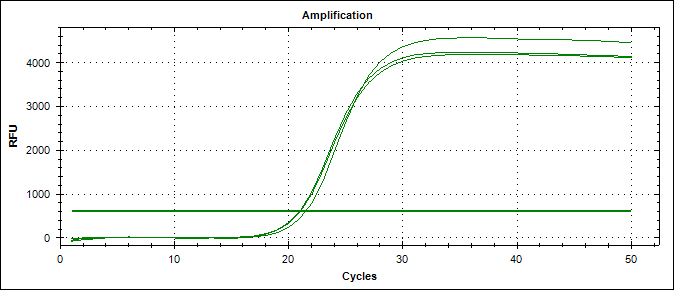


| Well | Target | Sample | Cq | Cq Mean | Cq Std. Dev |
| --- | --- | --- | --- | --- | --- |
| B01 | mir16 | ECWS35 | 21.52 | 21.21 | 0.269 |
| B02 | mir16 | ECWS35 | 21.02 | 21.21 | 0.269 |
| B03 | mir16 | ECWS35 | 21.09 | 21.21 | 0.269 |


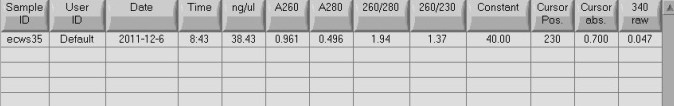


V ^ECWS35^= Sample ^ECWS35^Con /50-sample ^ECWS35^Ct_miR-16_

=38.43/28.79

=1.33484


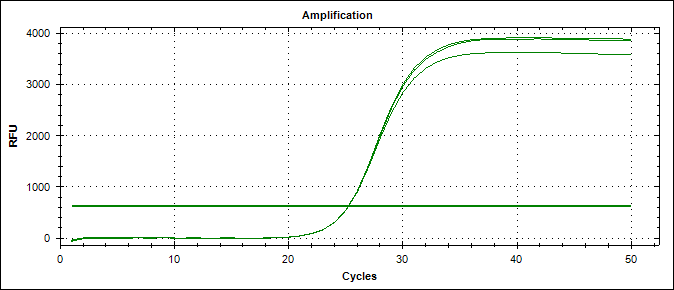


| Well | Target | Sample | Cq | Cq Mean | Cq Std. Dev |
| --- | --- | --- | --- | --- | --- |
| A04 | mir16 | ECWS36 | 25.24 | 25.23 | 0.021 |
| A05 | mir16 | ECWS36 | 25.24 | 25.23 | 0.021 |
| A06 | mir16 | ECWS36 | 25.20 | 25.23 | 0.021 |


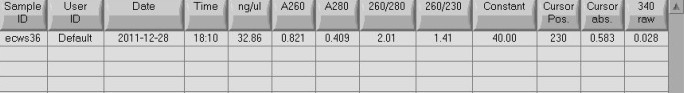


V ^ECWS36^= Sample ^ECWS36^Con /50-sample ^ECWS36^Ct_miR-16_

=32.86/24.77

=1.3266


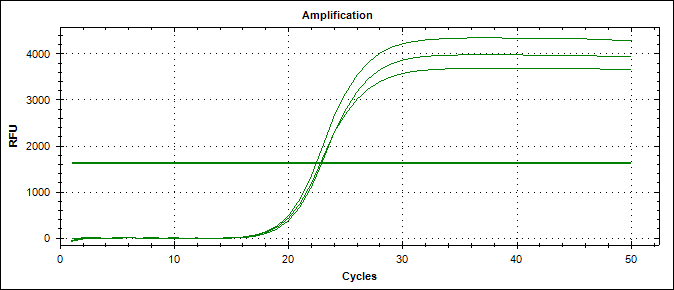


| Well | Target | Sample | Cq | Cq Mean | Cq Std. Dev |
| --- | --- | --- | --- | --- | --- |
| A04 | mir16 | ECWS37 | 22.90 | 22.70 | 0.251 |
| A05 | mir16 | ECWS37 | 22.41 | 22.70 | 0.251 |
| A06 | mir16 | ECWS37 | 22.78 | 22.70 | 0.251 |


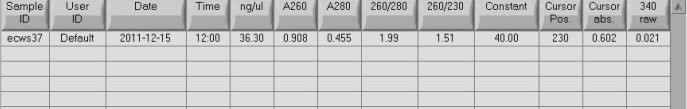


V ^ECWS37^= Sample ^ECWS37^Con /50-sample ^ECWS37^Ct_miR-16_

=36.3/27.3

=1.32967


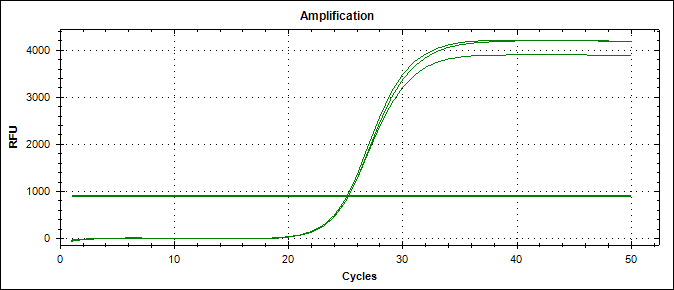


| Well | Target | Sample | Cq | Cq Mean | Cq Std. Dev |
| --- | --- | --- | --- | --- | --- |
| A04 | mir16 | ECWS38 | 25.24 | 25.20 | 0.072 |
| A05 | mir16 | ECWS38 | 25.24 | 25.20 | 0.072 |
| A06 | mir16 | ECWS38 | 25.11 | 25.20 | 0.072 |


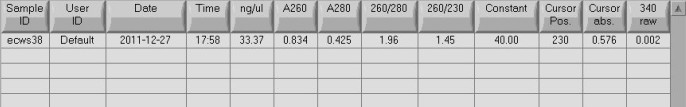


V ^ECWS38^= Sample ^ECWS38^Con /50-sample ^ECWS38^Ct_miR-16_

=33.37/24.8

=1.34556


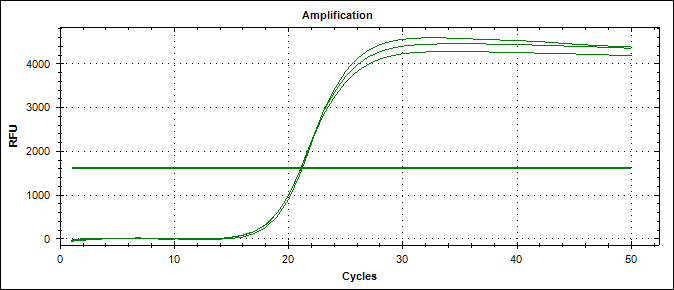


| Well | Target | Sample | Cq | Cq Mean | Cq Std. Dev |
| --- | --- | --- | --- | --- | --- |
| B01 | mir16 | ECWS39 | 21.22 | 21.14 | 0.069 |
| B02 | mir16 | ECWS39 | 21.11 | 21.14 | 0.069 |
| B03 | mir16 | ECWS39 | 21.09 | 21.14 | 0.069 |


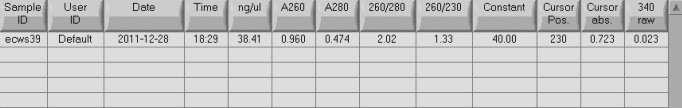


V ^ECWS39^= Sample ^ECWS39^Con /50-sample ^ECWS39^Ct_miR-16_

=38.41/28.86

=1.33091


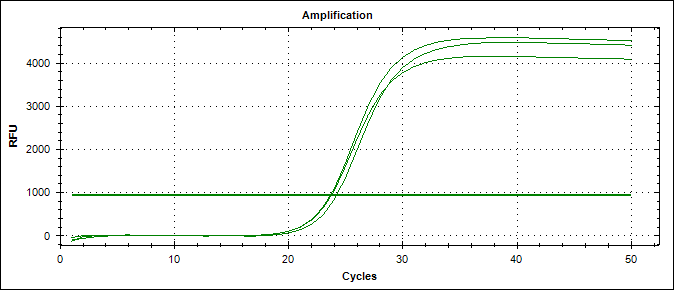


| Well | Target | Sample | Cq | Cq Mean | Cq Std. Dev |
| --- | --- | --- | --- | --- | --- |
| A01 | mir16 | NWS1 | 24.21 | 23.88 | 0.289 |
| A02 | mir16 | NWS1 | 23.77 | 23.88 | 0.289 |
| A03 | mir16 | NWS1 | 23.66 | 23.88 | 0.289 |


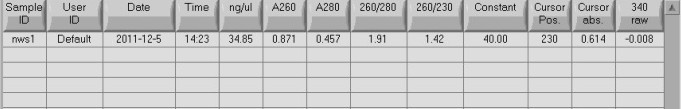


V ^NWS1^= Sample ^NWS1^Con /50-sample ^NWS1^Ct_miR-16_

=34.85/26.12

=1.33423


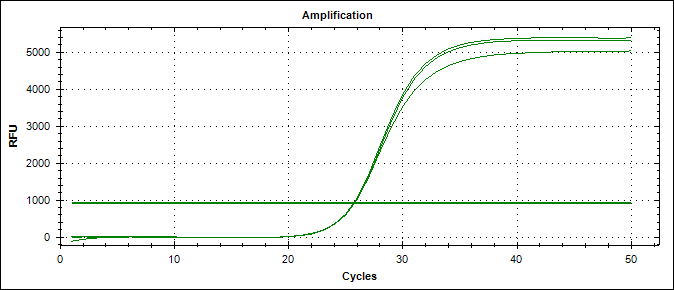


| Well | Target | Sample | Cq | Cq Mean | Cq Std. Dev |
| --- | --- | --- | --- | --- | --- |
| B01 | mir16 | NWS2 | 25.79 | 25.74 | 0.051 |
| B02 | mir16 | NWS2 | 25.69 | 25.74 | 0.051 |
| B03 | mir16 | NWS2 | 25.75 | 25.74 | 0.051 |


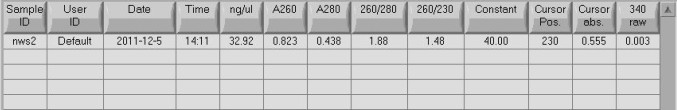


V ^NWS2^= Sample ^NWS2^Con /50-sample ^NWS2^Ct_miR-16_

=32.92/24.26

=1.35697


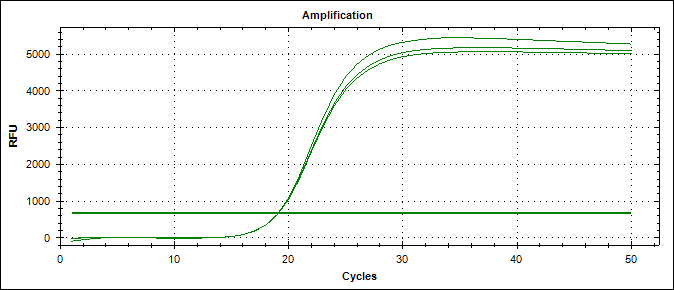


| Well | Target | Sample | Cq | Cq Mean | Cq Std. Dev |
| --- | --- | --- | --- | --- | --- |
| B01 | mir16 | NWS3 | 19.07 | 19.10 | 0.023 |
| B02 | mir16 | NWS3 | 19.10 | 19.10 | 0.023 |
| B03 | mir16 | NWS3 | 19.11 | 19.10 | 0.023 |


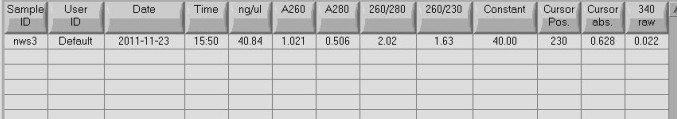


V ^NWS3^= Sample ^NWS3^Con /50-sample ^NWS3^Ct_miR-16_

=40.84/30.9

=1.32168


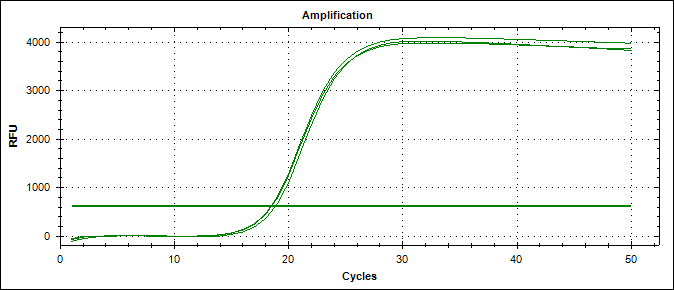


| Well | Target | Sample | Cq | Cq Mean | Cq Std. Dev |
| --- | --- | --- | --- | --- | --- |
| A01 | mir16 | NWS4 | 18.87 | 18.61 | 0.224 |
| A02 | mir16 | NWS4 | 18.51 | 18.61 | 0.224 |
| A03 | mir16 | NWS4 | 18.46 | 18.61 | 0.224 |


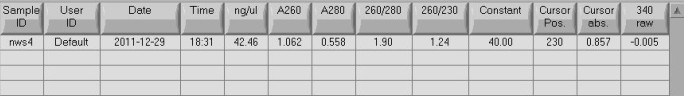


V ^NWS4^= Sample ^NWS4^Con /50-sample ^NWS4^Ct_miR-16_

=42.46/31.39

=1.35266


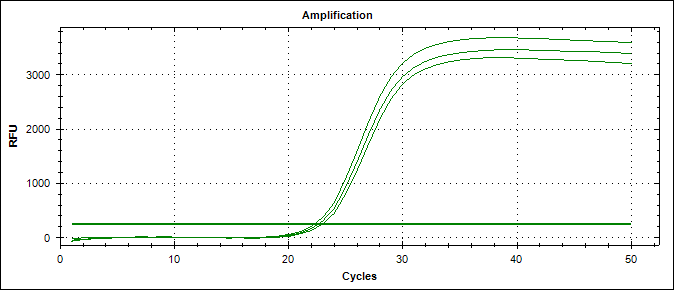


| Well | Target | Sample | Cq | Cq Mean | Cq Std. Dev |
| --- | --- | --- | --- | --- | --- |
| H01 | mir16 | NWS5 | 24.67 | 24.37 | 0.304 |
| H02 | mir16 | NWS5 | 24.37 | 24.37 | 0.304 |
| H03 | mir16 | NWS5 | 24.07 | 24.37 | 0.304 |


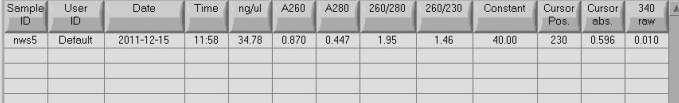


V ^NWS5^= Sample ^NWS5^Con /50-sample ^NWS5^Ct_miR-16_

=34.78/25.63

=1.357


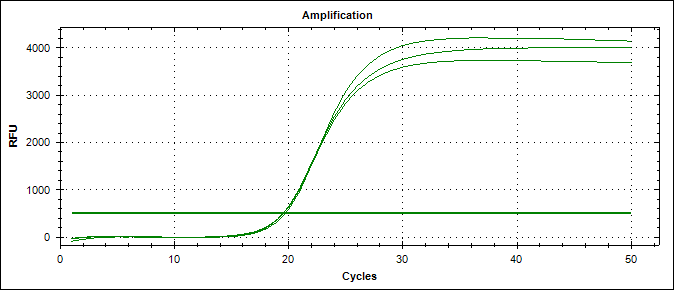


| Well | Target | Sample | Cq | Cq Mean | Cq Std. Dev |
| --- | --- | --- | --- | --- | --- |
| B01 | mir16 | NWS6 | 20.19 | 20.10 | 0.082 |
| B02 | mir16 | NWS6 | 20.08 | 20.10 | 0.082 |
| B03 | mir16 | NWS6 | 20.03 | 20.10 | 0.082 |


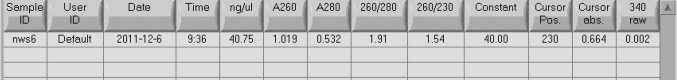


V ^NWS6^= Sample ^NWS6^Con /50-sample ^NWS6^Ct_miR-16_

=40.75/29.9

=1.36288


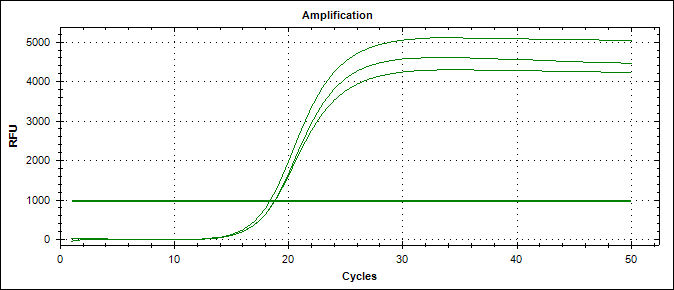


| Well | Target | Sample | Cq | Cq Mean | Cq Std. Dev |
| --- | --- | --- | --- | --- | --- |
| A01 | mir16 | NWS7 | 18.73 | 18.61 | 0.243 |
| A02 | mir16 | NWS7 | 18.77 | 18.61 | 0.243 |
| A03 | mir16 | NWS7 | 18.33 | 18.61 | 0.243 |


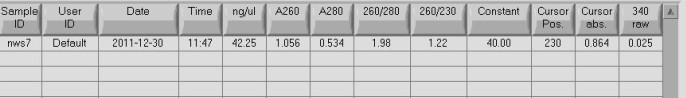


V ^NWS7^= Sample ^NWS7^Con /50-sample ^NWS7^Ct_miR-16_

=42.25/31.39

=1.34501


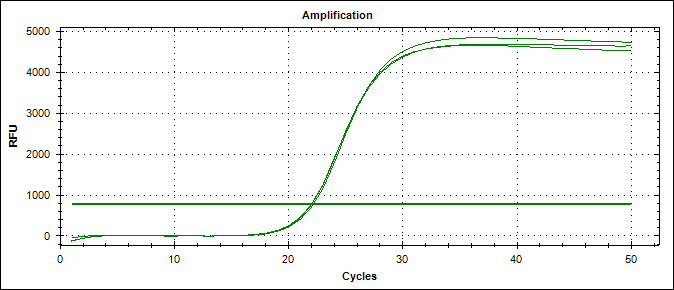


| Well | Target | Sample | Cq | Cq Mean | Cq Std. Dev |
| --- | --- | --- | --- | --- | --- |
| A01 | mir16 | NWS8 | 22.16 | 22.06 | 0.083 |
| A02 | mir16 | NWS8 | 22.03 | 22.06 | 0.083 |
| A03 | mir16 | NWS8 | 22.00 | 22.06 | 0.083 |


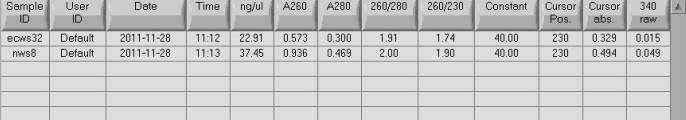


V ^NWS8^= Sample ^NWS8^Con /50-sample ^NWS8^Ct_miR-16_

=37.45/27.94

=1.34037


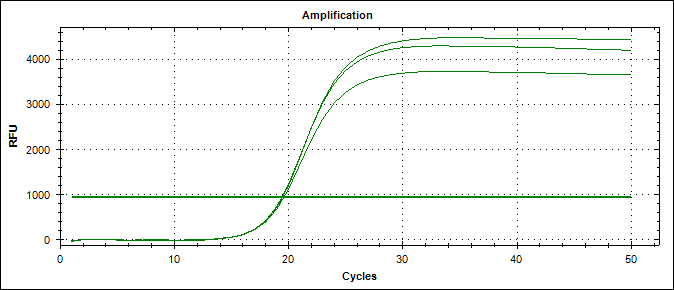


| Well | Target | Sample | Cq | Cq Mean | Cq Std. Dev |
| --- | --- | --- | --- | --- | --- |
| A04 | mir16 | NWS9 | 19.44 | 19.47 | 0.094 |
| A05 | mir16 | NWS9 | 19.58 | 19.47 | 0.094 |
| A06 | mir16 | NWS9 | 19.40 | 19.47 | 0.094 |


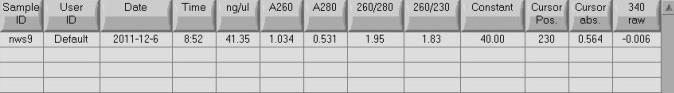


V ^NWS9^= Sample ^NWS9^Con /50-sample ^NWS9^Ct_miR-16_

=41.35/30.53

=1.35441


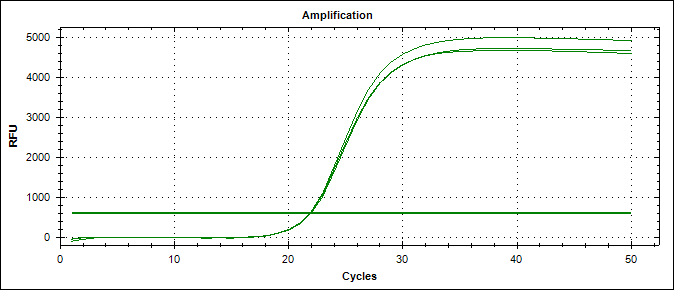


| Well | Target | Sample | Cq | Cq Mean | Cq Std. Dev |
| --- | --- | --- | --- | --- | --- |
| B01 | mir16 | NWS10 | 21.97 | 21.87 | 0.086 |
| B02 | mir16 | NWS10 | 21.84 | 21.87 | 0.086 |
| B03 | mir16 | NWS10 | 21.80 | 21.87 | 0.086 |


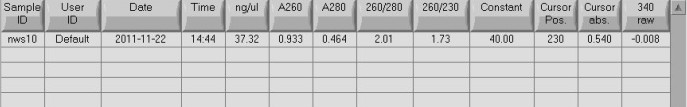


V ^NWS10^= Sample ^NWS10^Con /50-sample ^NWS10^Ct_miR-16_

=37.32/28.13

=1.3267


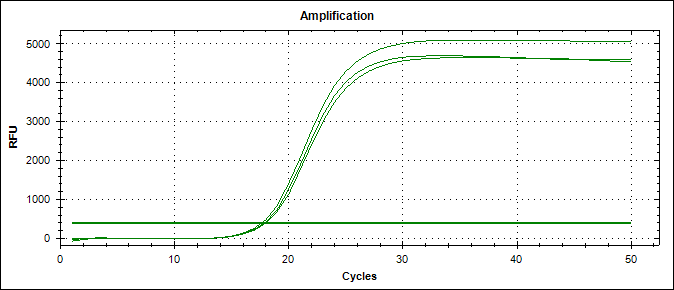


| Well | Target | Sample | Cq | Cq Mean | Cq Std. Dev |
| --- | --- | --- | --- | --- | --- |
| A01 | mir16 | NWS11 | 18.03 | 17.86 | 0.198 |
| A02 | mir16 | NWS11 | 17.91 | 17.86 | 0.198 |
| A03 | mir16 | NWS11 | 17.64 | 17.86 | 0.198 |


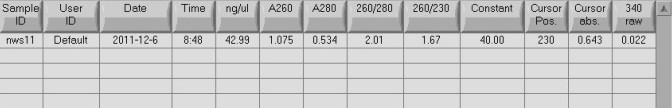


V ^NWS11^= Sample ^NWS11^Con /50-sample ^NWS11^Ct_miR-16_

=42.99/32.14

=1.33759

| Well | Target | Sample | Cq | Cq Mean | Cq Std. Dev |
| --- | --- | --- | --- | --- | --- |
| B01 | mir16 | NWS12 | 21.37 | 21.25 | 0.104 |
| B02 | mir16 | NWS12 | 21.17 | 21.25 | 0.104 |
| B03 | mir16 | NWS12 | 21.21 | 21.25 | 0.104 |

V ^NWS12^= Sample ^NWS12^Con /50-sample ^NWS12^Ct_miR-16_

=38.56/28.75

=1.34122

| Well | Target | Sample | Cq | Cq Mean | Cq Std. Dev |
| --- | --- | --- | --- | --- | --- |
| B01 | mir16 | NWS13 | 21.84 | 21.75 | 0.088 |
| B02 | mir16 | NWS13 | 21.66 | 21.75 | 0.088 |
| B03 | mir16 | NWS13 | 21.75 | 21.75 | 0.088 |

V ^NWS13^= Sample ^NWS13^Con /50-sample ^NWS13^Ct_miR-16_

=37.62/28.25

=1.33168

| Well | Target | Sample | Cq | Cq Mean | Cq Std. Dev |
| --- | --- | --- | --- | --- | --- |
| B01 | mir16 | NWS14 | 25.90 | 25.60 | 0.264 |
| B02 | mir16 | NWS14 | 25.40 | 25.60 | 0.264 |
| B03 | mir16 | NWS14 | 25.51 | 25.60 | 0.264 |

V ^NWS14^= Sample ^NWS14^Con /50-sample ^NWS14^Ct_miR-16_

=32.98/24.4

=1.35164

| Well | Target | Sample | Cq | Cq Mean | Cq Std. Dev |
| --- | --- | --- | --- | --- | --- |
| A01 | mir16 | NWS15 | 18.91 | 18.57 | 0.326 |
| A02 | mir16 | NWS15 | 18.53 | 18.57 | 0.326 |
| A03 | mir16 | NWS15 | 18.27 | 18.57 | 0.326 |

V ^NWS15^= Sample ^NWS15^Con /50-sample ^NWS15^Ct_miR-16_

=42.71/31.43

=1.35889

| Well | Target | Sample | Cq | Cq Mean | Cq Std. Dev |
| --- | --- | --- | --- | --- | --- |
| B01 | mir16 | NWS16 | 19.52 | 18.95 | 0.492 |
| B02 | mir16 | NWS16 | 18.63 | 18.95 | 0.492 |
| B03 | mir16 | NWS16 | 18.71 | 18.95 | 0.492 |

V ^NWS16^= Sample ^NWS16^Con /50-sample ^NWS16^Ct_miR-16_

=40.82/31.05

=1.31465

| Well | Target | Sample | Cq | Cq Mean | Cq Std. Dev |
| --- | --- | --- | --- | --- | --- |
| B01 | mir16 | NWS17 | 24.86 | 24.55 | 0.276 |
| B02 | mir16 | NWS17 | 24.43 | 24.55 | 0.276 |
| B03 | mir16 | NWS17 | 24.35 | 24.55 | 0.276 |

V ^NWS17^= Sample ^NWS17^Con /50-sample ^NWS17^Ct_miR-16_

=33.38/25.45

=1.31159

| Well | Target | Sample | Cq | Cq Mean | Cq Std. Dev |
| --- | --- | --- | --- | --- | --- |
| A04 | mir21 | NWS18 | 21.55 | 21.62 | 0.066 |
| A05 | mir21 | NWS18 | 21.67 | 21.62 | 0.066 |
| A06 | mir21 | NWS18 | 21.65 | 21.62 | 0.066 |

V ^NWS18^= Sample ^NWS18^Con /50-sample ^NWS18^Ct_miR-16_

=38.02/28.38

=1.33968

| Well | Target | Sample | Cq | Cq Mean | Cq Std. Dev |
| --- | --- | --- | --- | --- | --- |
| A01 | mir16 | NWS19 | 23.92 | 23.81 | 0.179 |
| A02 | mir16 | NWS19 | 23.90 | 23.81 | 0.179 |
| A03 | mir16 | NWS19 | 23.60 | 23.81 | 0.179 |

V ^NWS19^= Sample ^NWS19^Con /50-sample ^NWS19^Ct_miR-16_

**=**34.24/26.19

=1.30373
